# Circulating extracellular vesicle characteristics differ between men and women following 12 weeks of concurrent exercise training

**DOI:** 10.14814/phy2.16016

**Published:** 2024-05-02

**Authors:** Christopher K. Kargl, Adam J. Sterczala, Daniella Santucci, William R. Conkright, Kellen T. Krajewski, Brian J. Martin, Julie P. Greeves, Thomas J. O'Leary, Sophie L. Wardle, Amrita Sahu, Fabrisia Ambrosio, Bradley C. Nindl

**Affiliations:** ^1^ Neuromuscular Research Lab/Warrior Human Performance Research Center University of Pittsburgh Pittsburgh Pennsylvania USA; ^2^ Army Health and Performance Research, Army Headquarters Andover UK; ^3^ Division of Surgery and Interventional Science University College London London UK; ^4^ Norwich Medical School University of East Anglia Norwich UK; ^5^ Department of Physical Medicine and Rehabilitation University of Pittsburgh Pittsburgh Pennsylvania USA; ^6^ Department of Environmental and Occupational Health University of Pittsburgh Pittsburgh Pennsylvania USA; ^7^ Discovery Center for Musculoskeletal Recovery, Schoen Adams Research Institute at Spaulding Boston Massachusetts USA; ^8^ Department of Physical Medicine & Rehabilitation Harvard Medical School Boston Massachusetts USA

**Keywords:** exercise, extracellular vesicles, sex differences

## Abstract

Concurrent resistance and endurance exercise training (CET) has well‐studied benefits; however, inherent hormonal and genetic differences alter adaptive responses to exercise between sexes. Extracellular vesicles (EVs) are factors that contribute to adaptive signaling. Our purpose was to test if EV characteristics differ between men and women following CET. 18 young healthy participants underwent 12‐weeks of CET. Prior to and following CET, subjects performed an acute bout of heavy resistance exercise (AHRET) consisting of 6 × 10 back squats at 75% 1RM. At rest and following AHRET, EVs were isolated from plasma and characteristics and miRNA contents were analyzed. AHRET elevated EV abundance in trained men only (+51%) and AHRET‐induced changes were observed for muscle‐derived EVs and microvesicles. There were considerable sex‐specific effects of CET on EV miRNAs, highlighted by larger variation following the 12‐week program in men compared to women at rest. Pathway analysis based on differentially expressed EV miRNAs predicted that AHRET and 12 weeks of CET in men positively regulates hypertrophy and growth pathways more so than in women. This report highlights sex‐based differences in the EV response to resistance and concurrent exercise training and suggests that EVs may be important adaptive signaling factors altered by exercise training.

## 1 INTRODUCTION

Resistance exercise training (RET) has numerous health benefits highlighted by increased muscle strength and hypertrophy as well as improved bone health, capillarization, insulin sensitivity, and body composition (Roberts et al., 2023). While both men and women can undergo hypertrophy and strength increases following chronic RET, some differences are present between sexes. Overall, men have greater muscle mass and type II muscle fiber area while women may have greater relative increases in upper body strength following training (Nuzzo, 2023). Although absolute muscle mass gains are higher in men, percent changes do not appear to differ between the sexes (Roberts et al., 2020).

Factors secreted into the circulation following exercise by skeletal muscle fibers and other cell types, termed exerkines, are facilitators of local and systemic exercise adaptations. Acute and chronic RET alter circulating concentrations of multiple exerkines including growth factors, cytokines, and hormones such as growth hormone (GH), insulin‐like growth factor‐1 (IGF‐1), interleukin‐6 (IL‐6), brain‐derived neurotrophic factor (BDNF), irisin, sex steroids, and others (Chow et al., 2022; Greeves et al., 2023; Zunner et al., 2022). Sex‐based differences in exerkine responses exist, highlighted by greater IL‐6 and testosterone increases following acute exercise in men (Benini et al., 2015) and differential responses of IGF‐1 and GH at different timepoints following acute and chronic RET (Hatfield et al., 2021; Pierce et al., 2020).

Extracellular vesicles (EVs) continue to garner attention as important biomarkers and exerkines. EVs are a class of cell‐derived vesicles that can act as signaling factors that transport numerous RNAs, proteins, and lipids to neighboring and systemic tissues. Once internalized by a target cell, EV cargo can impact intracellular signaling and metabolism (Colombo et al., 2014). There are multiple EV subtypes—exosomes, microvesicles, and apoptotic bodies—and they are categorized according to size, release mechanisms, functions, and surface markers (Colombo et al., 2014; Nederveen et al., 2020). Immediately following acute endurance exercise, circulating and muscle‐derived EV release is increased (Brahmer et al., 2019; Frühbeis et al., 2015; Guescini et al., 2015) and contents are altered in support of exercise adaptations (Abdelsaid et al., 2022; Gao et al., 2021; Whitham et al., 2018).

Although most reports have studied endurance exercise, several studies have shown resistance exercise also alters EV structural and biochemical characteristics. Acute RET shifts the surface marker profile of circulating EVs (Conkright, Beckner, Sterczala, et al., 2022; Just et al., 2020), alters EV microRNA contents (Annibalini et al., 2019; Just et al., 2020), and induces release of EVs that promote cell growth in the muscle niche (Gu et al., 2022; Just et al., 2020). In mice, a supraphysiological hypertrophic stimulus prompts skeletal muscle to release EVs enriched with miRNA‐1 that are delivered to adipose tissue and induce lipolysis (Vechetti et al., 2021). The potential contribution of post‐RET EVs to muscle hypertrophy and related signaling pathway regulation (i.e., PI3K/Akt/mTOR) has not been analyzed. The impact of RET on EV release is not entirely clear, with some studies showing upregulation of EV release pathways in skeletal muscle (Garner et al., 2022) and increased circulating EV abundance (Annibalini et al., 2019) following acute resistance exercise while the majority show no impact of RT on total EV abundance (Barcellos et al., 2020; Conkright, Beckner, Sterczala, et al., 2022; Gu et al., 2022; Just et al., 2020).

Questions remain as to whether EV characteristics following exercise training differ between men and women, as the vast majority of exercise studies have been done in men. Previously our lab observed that following an acute bout of RET, men have an increase in circulating small EVs and muscle‐derived EVs although women proportionally release a greater amount of muscle‐derived EVs (Conkright, Beckner, Sterczala, et al., 2022). Additionally, physical activity guidelines recommend people participate in both resistance and endurance exercise; however, it is unknown whether long‐term concurrent training alters circulating EV characteristics, although 1 week of concurrent exercise is sufficient to alter resting EV contents (Sullivan et al., 2022). It is important to understand whether long‐term concurrent exercise training alters the signaling potential of circulating EVs as EVs have been shown to facilitate acute exercise adaptations. It is additionally vital to include both men and women in these studies to investigate if the EV signaling response to exercise training is sex‐specific. Due to differences in training methodology and scope of previous studies, we sought to determine the impact of 12 weeks of CET on circulating EV abundance, surface marker profile, and miRNA contents in young men and women at rest and after acute bouts of RET to determine if this novel exerkine is influenced by biological sex.

## 2 METHODS

### 2.1 These data were collected from a subset of subjects enrolled in a larger prospective cohort study aimed at optimizing physical readiness and training for military occupational performance in men and women (Conkright, Beckner, Sterczala, et al., 2022; Sterczala et al., 2023). Participants

Recreationally active and healthy men and women (18–36 years) were recruited to participate in this study and written informed consent was obtained from each subject before participating. Subjects were eligible if they participated in 30 min of physical activity at least three times per week and were free of any injuries that prohibited them from exercise training. Subjects were excluded if they were training for competitive sporting events, had a self‐reported weight fluctuation of ≥10 lbs. within the last 2‐months, had a chronic medical condition, had a BMI >35 kg/m^2^, were current or recent (6 moths) smokers, had suffered a musculoskeletal injury within the past 2 years that precluded physical activity for more than a month, were pregnant or planning to become pregnant, or had been taking hormone affecting medications, with the exception of oral contraceptives. A more thorough detailing of subject exercise history and study criteria has been described previously (Sterczala et al., 2023).

Subject subsets were randomly selected from the larger cohort (*n* = 39) for EV amounts, subpopulation, and content analyses. Eighteen subjects (*n* = 9 men and women) were selected for analysis of EV abundance and subpopulation proportions, and from that cohort 10 subjects (*n* = 5 men and women) were utilized for additional miRNA content analyses. These sample sizes were selected based on previous exercise EV studies (Conkright, Beckner, Sahu, et al., 2022; Karvinen et al., 2023). Four time points were analyzed per subject and all subjects completed each time point and had sufficient plasma for each analysis. There were no differences in demographic data (height, weight, body mass index [BMI] and, body fat %) between the subject subsets and the larger cohort population. Body composition, including lean and fat mass, was assessed via dual‐energy X‐ray absorptiometry (DXA) using the Lunar iDXA (GE Healthcare, IL, USA).

### 2.2 Experimental design

#### 2.2.1 Acute resistance exercise bout

Subjects performed an acute heavy resistance exercise test (AHRET) at least 5 days after 1‐RM testing prior to and following the 12‐week exercise training intervention. Following a dynamic warmup, subjects completed the AHRET which consisted of six sets of 10 repetitions of back squats. Loading began at 75% of each individual's 1‐RM and was decreased as necessary for participants to complete all repetitions, with 2‐min rests between sets. The AHRET is a well‐established resistance exercise protocol for inducing hormonal and stress responses (Nindl et al., 2001; Pierce et al., 2020). All sessions were supervised by a Certified Strength and Conditioning Specialist (CSCS) and trained spotters were present to ensure safety. The weight was increased or decreased if the RPE on the previous set was ≤4 or 10, respectively, or at the discretion of the CSCS if the RPE was an 8 or 9 on the previous set to ensure maximal effort. All acute resistance bouts took place in the morning and prior to testing, participants fasted overnight and withheld from caffeine for 8 h, non‐steroidal anti‐inflammatory drug use and exercise for 72 h.

#### 2.2.2 Exercise training intervention

**FIGURE 1:**
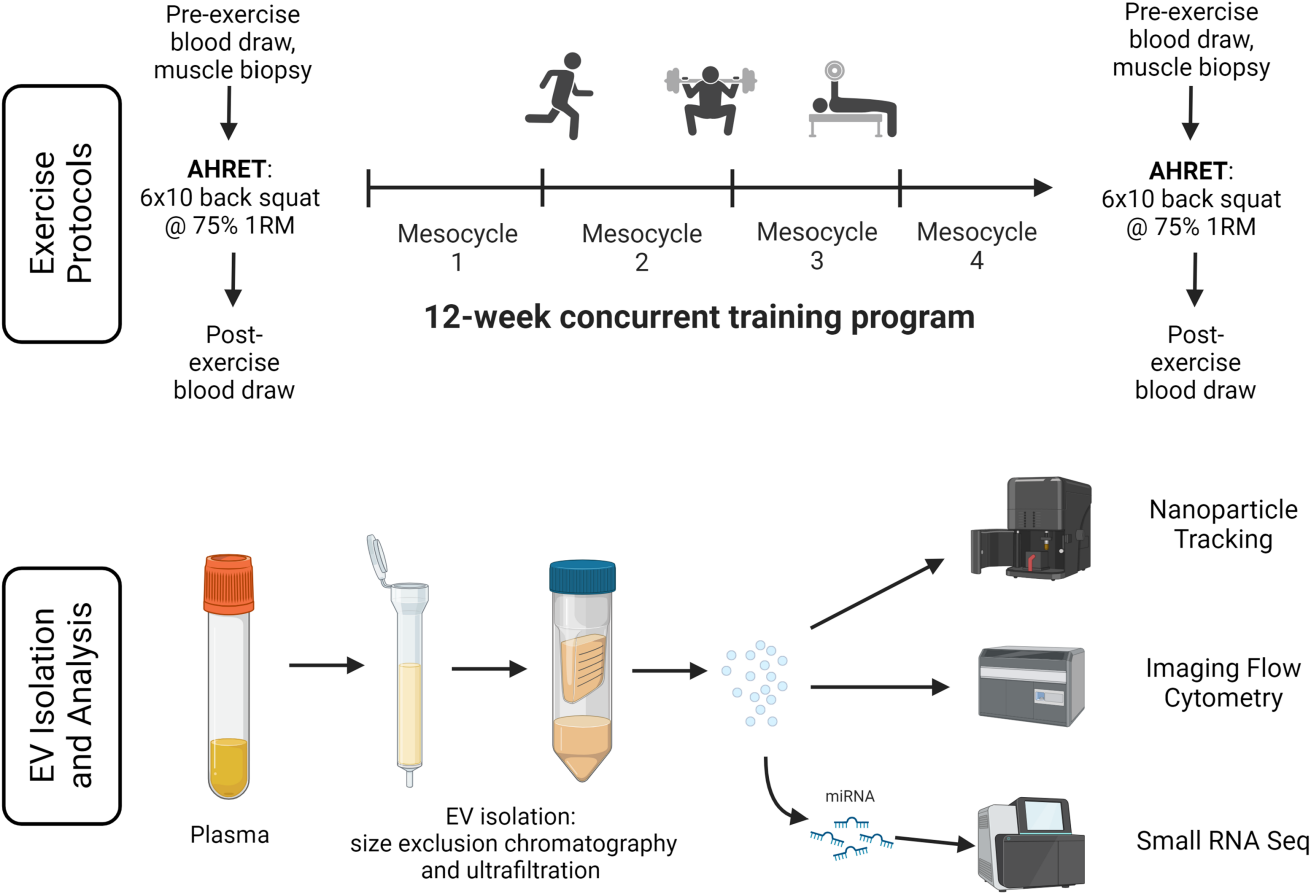
Schematic overview of study methodology. Prior to the chronic training program subjects had blood draws taken before and after an acute bout of heavy resistance exercise training (AHRET, acute bout of heavy resistance exercise; 6 × 10 at 75% 1 RM on the back squat). Subjects then underwent 12 weeks of resistance exercise training, following which they repeated the AHRET bout. Extracellular vesicles (EVs) were isolated from plasma samples via a combined size exclusion chromatography and ultrafiltration technique (SEC‐UF). EV characteristics and contents were analyzed via Nanoparticle Tracking Analysis, Imaging Flow Cytometry, and small RNA sequencing. Figure generated using BioRender.

A schematic overview of the experimental design is depicted in Figure 1. Subjects completed a 12‐week periodized concurrent resistance and interval training program as described in detail previously (Sterczala et al., 2023). Briefly, the training program consisted of 4 mesocycles: general physical preparedness, preparation for peak force production, peak force development, and rate of force development. The second, third and fourth mesocycles were followed by a 1‐week deload, during which participants performed exercises in the same repetition ranges but at approximately 50% of the loading intensity used in previous weeks. During the third visit of each deload week, new exercise 1‐RMs were determined for the subsequent mesocycle. The duration, intensities, and set and repetition ranges are displayed in Table S1. Specific exercise varied throughout the intervention but focused on complex, multi‐joint movements such as deadlifts, squats, jumps, and loaded carries. Interval training varied during the 12‐weeks and was based on age‐predicted maximal heart rate (HR_max_ = 220−age) and consisted of runs/sprints, bodyweight plyometrics, and loaded carries. During the first and second mesocycle, interval training was performed at 70–85% HR_max_ and above 80% HR_max_ during the final two mesocycles. During the intervention, the participants completed three 60–90‐min training sessions per week for a total of 36 sessions. Each session started with a dynamic warmup followed by resistance training and ended with interval training which included variations of sprints, jumps, and loaded carries. Participants were required to maintain a minimum of 95% training session compliance. VO_2_peak was assessed before and after the 12‐week training intervention via an incremental Bruce treadmill graded exercise test on a treadmill. The test was performed fasted following a 24 h minimum rest period, participants performed to volitional failure with oxygen consumption measured via indirect calorimetry (Parvo TrueOne). Expired gases were sampled and analyzed breath by breath. To be considered as having reached a valid VO_2_peak at least two of the following conditions had to be met: a plateau in oxygen consumption despite an increase in intensity, maximal heart rate within 10 beats per minute of the participant's age‐predicted max, and a respiratory exchange ratio ≥1.15.

### 2.3 Blood draw and plasma EV isolation

Blood was drawn at four different timepoints throughout the study, before and immediately following the AHRET at baseline and following the 12‐week exercise training protocol. Participants fasted overnight prior to morning blood draws. Blood was drawn from an upper arm vein into 6 mL EDTA plasma tubes using a 21‐ or 23‐gauge needle (Becton, Dickinson and Company Vacutainer, Franklin Lakes, NJ, USA). Plasma tubes were centrifuged at 1500×*g* for 15 min at 4°C and plasma was isolated and stored at −80°C. Plasma samples with signs of hemolysis were not used in the study. Plasma samples were thawed over ice and EVs were isolated via size exclusion chromatography (SEC; Generation 2 qEV1 70 nm columns; IZON, Medford, ME, USA) according to the manufacturer's instructions. Briefly, 1 mL of plasma was loaded and ran through the SEC column with sterile‐filtered PBS and the EV fraction was collected. To elevate EV concentration for subsequent analyses, the 2.8 mL EV fractions were added to 2–6 mL Protein concentrator tubes with 50 kDa filters (Thermo Fisher Scientific, Waltham, MA, USA) and centrifuged at 3500 x g to a final volume of 200 μL or 1 mL, depending on the analysis of interest. EVs were stored at −80°C until further analysis.

### 2.4 RNA isolation

Total RNA was isolated from EV samples using the Plasma/Serum Exosome Purification and RNA isolation Mini Kit (Norgen Biotek Corp, Thorold, ON, Canada) according to the supplementary protocol for RNA purification from previously isolated exosome samples provided by the manufacturer. A final volume of 15 μL was collected in manufacturer provided elution buffer and stored at −80°C prior to quality control testing. Small RNA size, purity, and concentration were determined prior to sequencing via a small RNA analysis kit on a 2100 Bioanalyzer System (Agilent Technologies, Santa Clara CA).

### 2.5 Small RNA sequencing

EV small RNA library preparation and sequencing was carried out by the Center for Medical Genomics at Indiana University School of Medicine. Single‐end libraries were constructed from 5 ng of EV RNA using QIAseq miRNA library kit (Qiagen, Redwood City, CA) according to the manufacturer's protocol and recommendations for low yield samples. Small RNA sequencing was performed using an Illumina NextSeq 500 system with HO 75 cycle flow cells. Quality control of the raw sequence data was done with FASTQC (Illumina version 0.11.5), with no trimming required. The raw sequencing data was analyzed by the Bioinformatics Core at Indiana University School of Medicine. Reads were mapped to the designated reference genome (miRbase_v22) using the Qiagen GeneGlobe RNA‐seq analysis portal (Version 3.0.1) with default parameters for miRNA analysis. Differential gene expression analysis was performed using the edgeR workflow as previously described (Robinson et al., 2010). Complete sequencing data is available to access via the Gene Expression Omnibus (GEO) database (accession # GSE232700). Differential gene expressions were generated for eight different comparisons. The comparisons were: resting vs post‐AHRET at baseline and following chronic training, resting at baseline vs resting following chronic training, and post‐AHRET at baseline vs post‐AHRET following chronic training. Each comparison was done in both men and women.

### 2.6 Biological pathway analysis

Biological pathways targeted by differentially expressed miRNAs were analyzed via DIANA mirpath v.3 and Ingenuity Pathway Analysis software (IPA; Qiagen). For all analyses, differentially expressed miRNAs between each of the eight individual comparisons were run through DIANA mirpath and IPA. Certain pathways related to cancer, infections, and non‐relevant disease states were excluded from analysis to focus on pathways relevant to our experimental design. DIANA mirpath generated general KEGG pathways and cellular functions targeted by differentially expressed miRNAs. IPA was utilized to identify specific biological pathways targeted by the miRNA contents and to establish visualization and directionality of pathway regulation. The miRNA target filter within IPA was utilized to generate miRNA: mRNA target interactions across multiple bioinformatics databases. A conservative approach was used for the mRNA target prediction where only experimentally observed targets were selected as determined via miRTarBase and TargetScan, respectively (Bartel, 2009; Hsu et al., 2011). A table containing the number of differentially expressed miRNAs and subsequent number of targeted mRNAs is included in Table S2. Complete listing of the differentially expressed miRNAs and the miRNA target filter output of all targeted mRNAs for each of the eight comparisons are in Files S1 and S2, respectively. Directionality of the targeted mRNAs was established by reversing the fold change of the miRNA that corresponded to each miRNA: mRNA interaction. Pathway analysis was then run on the targeted mRNAs to generate canonical pathways predicted to be regulated significantly and differentially between each comparison. Additionally, IPA's Function analysis capability was utilized to determine how the differentially expressed miRNAs were predicted to regulate different physiological functions. Heat maps and Venn diagrams were created using the ClustVis webtool (https://biit.cs.ut.ee/clustvis/).

### 2.7 Nanoparticle tracking analysis

Nanoparticle tracking analysis (NTA) was utilized to measure EV amount. Briefly, a small portion of the EV sample was diluted 100× with sterile‐filtered PBS to a final volume of 1 mL. EV particle concentration and size were characterized using a NS300 NanoSight instrument (Malvern Panalytical, Westborough, MA, USA) equipped with a 532 nm laser and a sCMOS camera. Samples were diluted 100× in sterile‐filtered PBS, loaded into a luer lock syringe and manually pumped into the chamber. The camera level was set to 10 for all sample captures and detection threshold was set to 7 for all sample analyses. Three 60‐s videos were captured for each sample and analyzed using NTA software (Malvern Panalytcial) to measure particle concentration/mL and particle size.

Vesicle abundance was further analyzed by measuring total protein concentration using a Pierce BCA kit (Thermo Fisher Scientific) according to the manufacturer's instructions. All EV amounts are reported per ml of plasma.

### 2.8 Imaging flow cytometry

The isolated EV fraction was analyzed using an Image Stream X Mark II imaging flow cytometer (EMD, Millipore Sigma, Seattle, WA). Protein surface markers for different EV subtypes were utilized to confirm presence and estimate subpopulation proportions of the circulating EVs. Proteins chosen for analysis represent markers of exosomes (CD9), microvesicles (vesicle‐associated membrane‐3 (VAMP3)), and apoptotic bodies (thrombospondin‐1 [THSD1]; Akers et al., 2013; Conkright, Beckner, Sahu, et al., 2022). α‐Sarcoglycan (SGCA) has also been implicated as a marker of muscle‐derived EVs. 250 μL of isolated EVs were added to 50 μL blocking buffer (3% BSA in sterile‐filtered PBS) and placed on a rocker to block for 1 h. at RT. Samples were then stained with the following antibodies and dilutions: anti‐human CD9 Alexa Fluor 700 (1:300 dilution; Novus Biologicals, CO), anti‐human VAMP3 Alexa Fluor 405 (1:300; Novus Biologicals), anti‐human thrombospondin (THSD‐1) Alexa Fluor 594 (1:100; Novus Biologicals), alpha sarcoglycan (SGCA) FITC (1:400; Biorbyt, St Louis, MO). Samples were incubated overnight at 4°C and run through the flow cytometer the following day. Several control conditions were utilized to establish proper gating, to correct for fluorescence carryover, and to ensure quality data was obtained. Briefly, buffer only and antibody only controls were run and single‐stained compensation controls (UltraComp eBeads Plus; Invitrogen, Waltham, MA) were used to correct for fluorescence carryover between channels. Fluorescence minus one (FMO) controls were run to establish final gating strategies for the fluorescent channels. All controls were prepared according to the staining protocol above, except for the compensation controls, which were prepared according to the manufacturer's instructions.

ImageStream settings were consistent for all samples: 60× magnification, high gain mode, fluidics set to high sensitivity and slow speed, auto‐focus, and auto‐centering. Laser voltages were adjusted to keep Raw Max pixels below 4e^3 and were as follows: 405 nm 150 mW, 488 nm 125 mW, 561 nm 150 mW, 642 nm 150 mW, and SSC 70 mW. All timepoints for a single subject were prepared and analyzed on the cytometer on the same day and each sample was run for 3 min.

Flow cytometry data were analyzed using the IDEAS 6.2 software (MilliporeSigma, Burlington, MA) as previously described (Conkright, Beckner, Sterczala, et al., 2022) with slight modifications. ImageStream SpeedBeads were gated out of all samples using a histogram of the side scatter channel and only gating events less than the high side scatter peaks indicative of single and clumped SpeedBeads. Quantification of scatter and fluorescent intensities was established using a masking strategy described by Tertel et al. (2020) optimized for EV analyses. Positive events in each EV subpopulation were then gated according to their appropriate fluorescent channel and the gates were adjusted using each FMO controls when appropriate. Template files were utilized so that all samples run on the same day were gated in the same manner. Batch analysis was done using the appropriate templates and a final statistical report was generated for each sample containing percentage gated of the EV events gate.

### 2.9 Muscle biopsies and immunoblotting

Muscle biopsies were performed as part of the larger study and utilized to examine Akt content before and after concurrent training. Biopsies were taken from the vastus lateralis on AHRET testing days using the modified Bergstrom needle technique. Visible fat and connective tissue was removed from muscle samples and samples were flash frozen in liquid nitrogen and stored at −80°C for future analysis. For immunoblotting, frozen muscle was homogenized using a Bullet Blender Tissue Homogenizer (Next Advance, BBX24) at 4°C in T‐PER tissue lysis buffer (Thermo Fisher Scientific) supplemented with HALT protease and phosphatase inhibitor cocktail (Thermo Fisher Scientific). After homogenization, lysates were centrifuged at 14,000 *g* for 5 min at 4°C. Supernatants protein concentration was quantified using a bicinchoninic acid (BCA) protein assay (Pierce; Thermo Fisher Scientific) and stored at −20°C. Loading samples were prepared using supernatants and Laemmli sample buffer (Bio‐Rad Laboratories, Hercules, CA) with 2‐mercaptoethanol then heated at 95°C for 15 min. 50 μg of each sample was loaded into 4%–15% Mini‐PROTEAN TGX Stain‐Free Protein gels (Bio‐Rad) and electrophoresis was run at 70 V for 15 min, followed by 130 V for 50 min. Gels were transferred to LF‐PVDF membrane (Bio‐Rad) for 7 min at 2.5A on a Trans‐Blot Turbo Transfer System (Bio‐Rad). Membranes were blocked for 5 min using EveryBlot Blocking Buffer (Bio‐Rad) at RT with gentle rocking. Immunoblotting was performed overnight at 4°C using an antibody for total‐Akt (Cell Signaling Technology, 2920). The next day, membranes were washed five times for 5 min with tris‐buffered saline with 0.1% Tween 20 (TBS‐T). Membranes were incubated for 1 h at RT with secondary antibodies, StarBright Blue 520 Goat Anti‐Mouse IgG (Bio‐Rad, 12005867). Membranes were washed five times for 5 min, air dried and imaged using a ChemiDoc MP (Bio‐Rad). Images were quantified using Image Lab software from Bio‐Rad and normalized to stain‐free total protein.

### 2.10 Statistical analysis

Data are presented as means ± SD, unless otherwise noted. Normality was confirmed via a Shapiro–Wilks test. Subject characteristics and Akt content between groups were analyzed with Student's *t*‐test. Unless previously detailed, other comparisons were analyzed using three‐way [Sex (2) × AHRET (2) × chronic training status (2)] repeated‐measures analysis of variance (ANOVA). Following a significant *F* ratio, Fisher's LSD post‐hoc analysis was performed on the significant interaction. Unless otherwise noted, significance was established at *p* ≤ 0.05 and Data were analyzed using GraphPad Prism version 9.4.

## 3 RESULTS

Demographic, body composition, and AHRET bout information for all subjects are displayed in Table 1, characteristics for the subset of subjects that had EV miRNA sequencing performed are displayed in Table S3. For both sets of subjects, there was no significant age difference between men and women. Women were shorter than men, had lower body and total lean mass, and higher body fat % than men both prior to and following the 12‐week CET. Men had greater squat 1‐RM and lifted more weight cumulatively during the AHRET bout than women at baseline and after CET. Following CET, both men and women gained body mass and lean mass but maintained body fat % compared to baseline. AHRET intensity level per set (% 1‐RM) was not impacted by sex or time. The 12‐week CET elevated squat 1‐RM and cumulative weight lifted during the AHRET bout, compared to baseline in both men and women. There was no impact of the 12‐week CET on VO_2_peak, men had greater peak VO_2_ than women both before and after CET.

**TABLE 1:** Subject characteristics (*N* = 9).

	Men (*n* = 9)		Women(*n* = 9)	
	Baseline	12 weeks	Baseline	12 weeks
Age (years)	27.3 ± 3.9 (19–32)		27.4 ± 3.8 (24.0–36.0)	
Height (cm)^a^	178.4 ± 4.2 (170.1–183.4)		165.2 ± 6.5 (155.4–174.8)	
Body mass (kg)^a^	88.1 ± 12.9 (76.9–92.6)	88.5 ± 11.9 (77.1–93.6)	66.1 ± 8.6 (52.3–79.5)	67.2 ± 9.2^b^ (53.9–78.27)
Body mass index (BMI; kg/m^2^)^a^	27.7 ± 3.8 (23.7–33.4)	27.8 ± 3.5 (25.1–33.3)	24.2 ± 3.2 (21.2–30.9)	24.7 ± 3.7 (21.5–32.7)
Body fat (%)^a^	24.5 ± 7.1 (14–36)	23.5 ± 6.1 (15–34)	31.0 ± 6.2 (25.2–44)	30.4 ± 7.0 (23.8–44)
Total lean mass mass (kg)^a^	63.4 ± 5.8 (55.4–73.3)	65.4 ± 5.9^b^ (57.4–75.6)	43.2 ± 5.9 (37.0–54.0)	44.8 ± 5.4^b^ (39.2–53.4)
Squat 1 RM (kg)^a^	123.3 ± 25.1 (100–170.5)	136.5 ± 30.9^b^ (100–177.3)	61.3 ± 10.8 (38.7–68.2)	79.3 ± 11.6^b^ (61.4–95.5)
VO_2_ peak (mL/kg × min; Men *n* = 7, Women *n* = 5)^a^	49.7 ± 6.4 (37.3–56.7)	49.2 ± 7.0 (37–58.2)	45.5 ± 5.5 (36.4–50.1)	43.2 ± 4.9 (36.1–48.8)
AHRET intensity/set (% 1 RM)	73.6 ± 4.2 (66–78.4)	74.6 ± 3.1 (68.4–79.3)	75.9 ± 6.4 (66.8–89.1)	73.4 ± 2.4 (68.4–75.4)
AHRET total weight (weight × reps × sets; kg)^a^	5415.6 ± 935.3	6075.6 ± 1217.2^b^	2772.2 ± 457.9	3490.0 ± 497.9^b^

*Note*: Data are presented as mean ± SD (range). Significance was set at *p* < 0.05.

^a^ A significant difference between men and women.

^b^ A significant difference between pre‐ and post‐training.

### 3.1 Effects of acute and chronic exercise on circulating EV abundance

**FIGURE 2:**
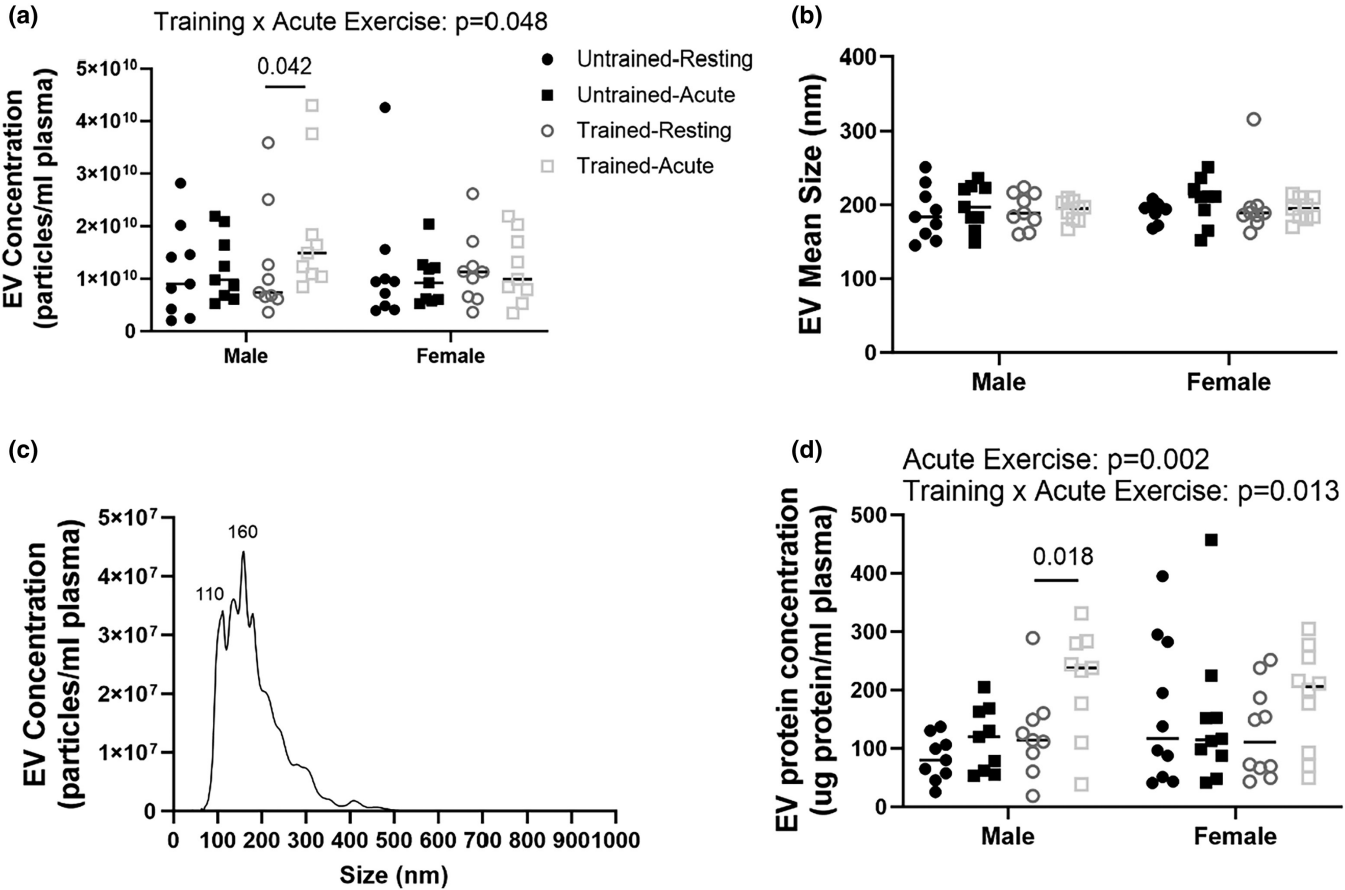
Acute exercise elevates EV release in trained men but not women. EV particle concentration (particles/mL plasma) and mean size (nm) as measured by Nanoparticle Tracking Analysis (NTA; a and b). Representative image of EV particle size distribution as measured by NTA (c). Protein concentration of isolated EVs as measured by BCA assay (d). Concentration data are displayed per mL of plasma used for EV isolation. *N* = 9. Statistical testing was done via three‐way ANOVA.

Circulating EV abundance was analyzed in two separate ways, as recommended by the International Society of Extracellular Vesicles (Théry et al., 2018), via NTA and through measuring total protein concentration of the EV fraction. There was a significant interaction of chronic training and acute exercise on NTA particle concentration (training × acute exercise: *p* = 0.048; Figure 2A), with acute exercise elevating circulating EVs after chronic exercise training but not at baseline in men (*p* = 0.042). EV mean size as measured by NTA did not differ between men and women and was not impacted by acute or chronic training (Figure 2b), representative image of EV size distribution from NTA shown in Figure 2c. EV protein concentration as measured by BCA assay showed similar effects of exercise on EV abundance as there was a significant interaction of chronic training and acute exercise (training × acute exercise: *p* = 0.0133; Figure 2d). In addition, there was a main effect of acute exercise on EV protein concentration (Resting: 124.71, Acute: 166.40; *p* = 0.002). Post‐hoc analysis was consistent with the NTA data, in that EV protein concentration following acute exercise was elevated following chronic exercise training but not at baseline in men (*p* = 0.018).

### 3.2 Effects of acute and chronic exercise on circulating EV subpopulations

**FIGURE 3:**
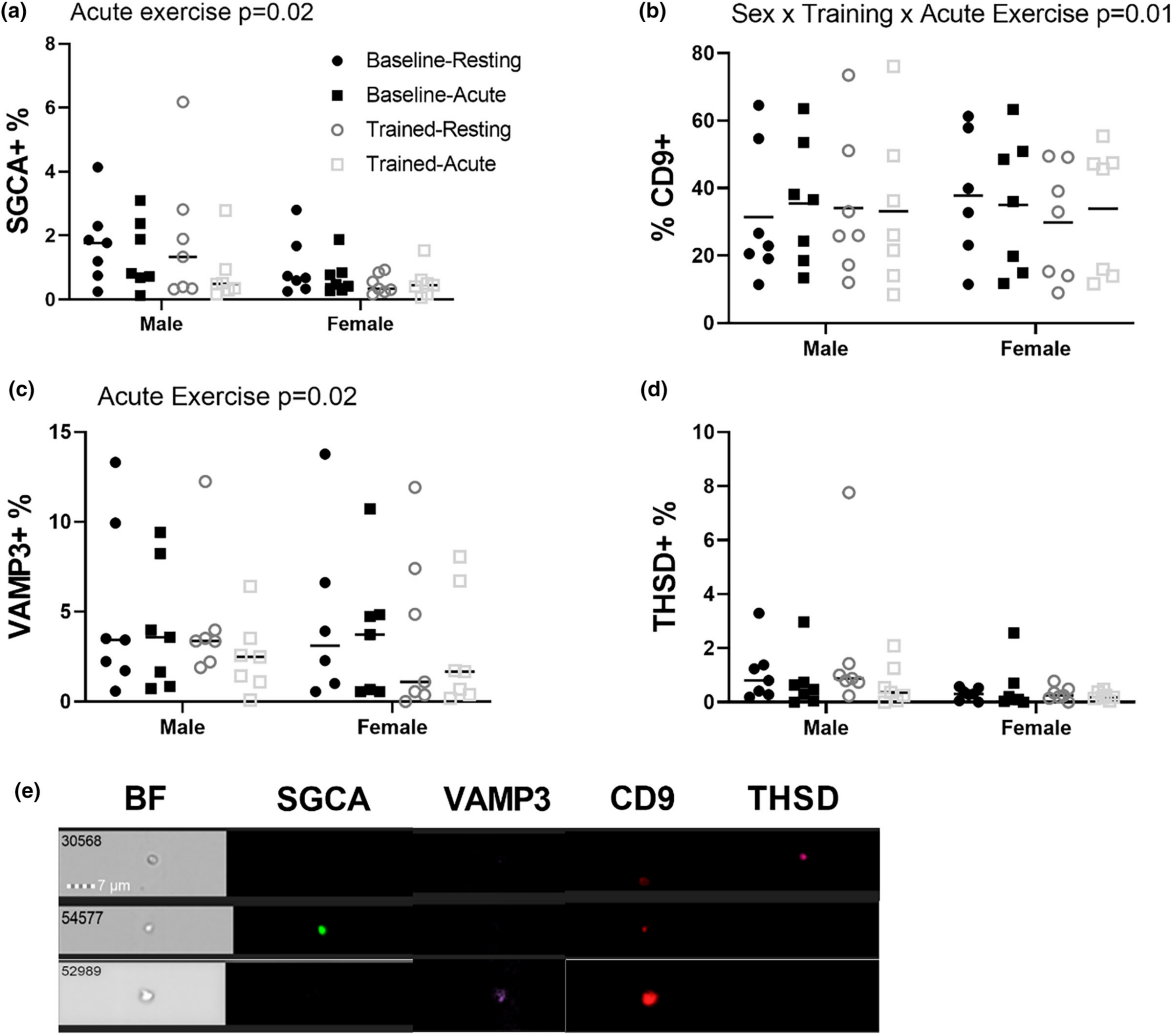
EV surface marker expression is altered by AHRET in men and women. The proportion of EVs expressing SGCA (a), CD9 (b), VAMP3 (c), and THSD (d) were measured via Imaging Flow Cytometry and are shown as percentage of total gated EVs. Representative images of EV particles with bright‐field (BF) image of each particle and subsequent fluorescent channel images showing presence or absence of muscle‐derived EVs (SGCA+), microvesicles (VAMP3+), exosomes (CD9+) and apoptotic bodies (THSD+) vesicles are shown (e). *N* = 9. Statistical testing was done via three‐way ANOVA.

EV subpopulation percentages by surface marker were evaluated using Imaging Flow Cytometry. AHRET resulted in a 33% decrease in the proportion of muscle‐derived SGCA + EVs (Figure 3A) among the circulating EV population, independent of sex and chronic training status (*p* = 0.023). For proportion of CD9+ EVs (Figure 3b), there was an interaction effect of biological sex, chronic training, and AHRET (*p* = 0.019), such that men had a slight increase following acute exercise before training and a slight decrease following training, with opposite trends occurring in women. There was also a main effect of AHRET on microvesicles (Figure 3c), as a 27% decrease in proportion of VAMP3^+^ microvesicles occurred following AHRET (*p* = 0.022). The proportion of THSD‐1+ apoptotic bodies (Figure 3d) was not impacted by biological sex, AHRET, or chronic training, although there was a trend towards higher THSD‐1+ EVs in men compared to women, independent of other variables (*p* = 0.07).

### 3.3 Effects of acute and chronic exercise on circulating EV miRNA contents

**FIGURE 4:**
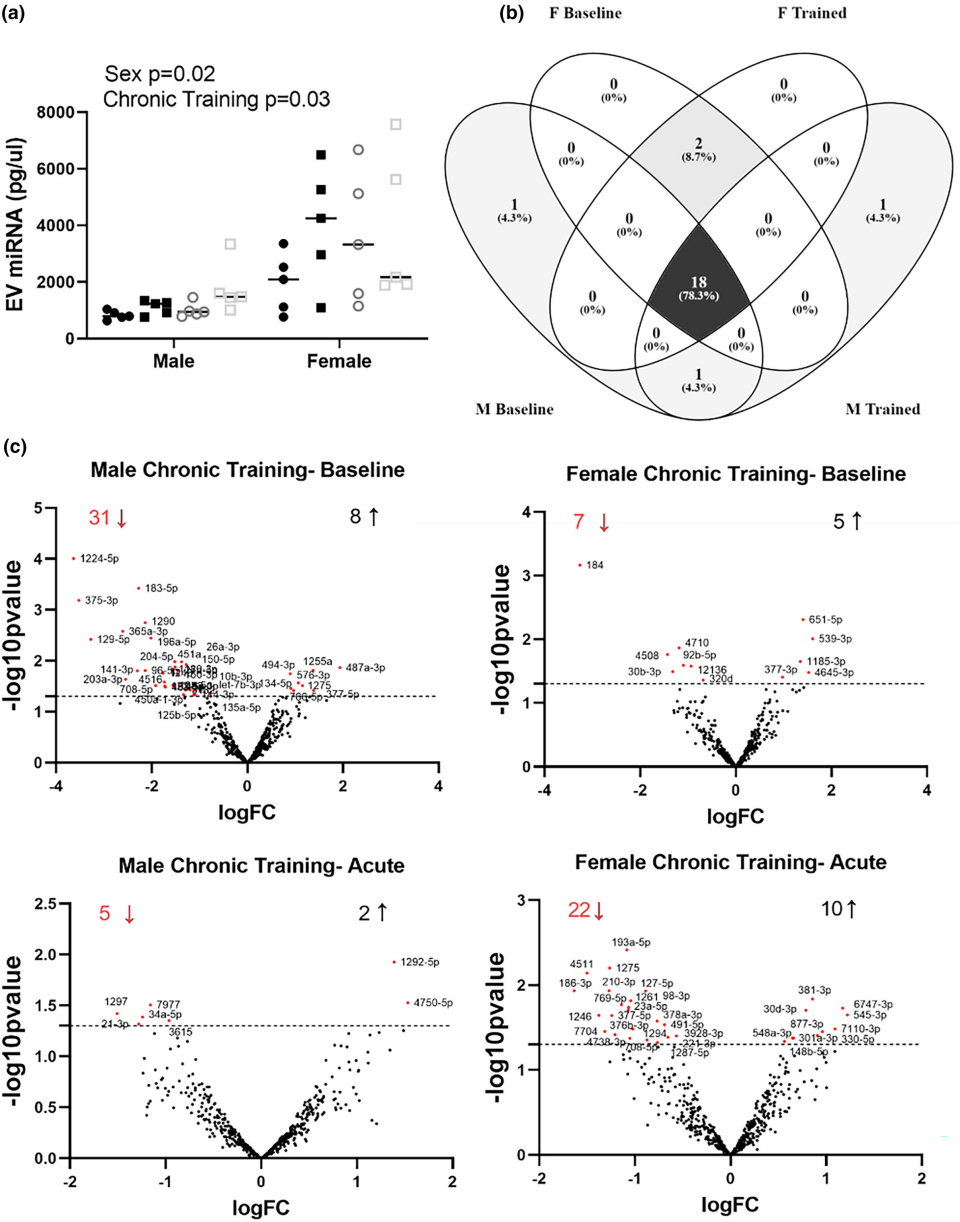
Sex‐based differences in EV miRNA contents in response to chronic exercise training. EV miRNA contents at each timepoint as measured via Bioanalyzer (a). Venn diagram depicting the 25 most enriched miRNAs contained within plasma‐EVs, before and after training in resting men and women (b). Volcano plots of statistical significance (*p*‐value) versus magnitude of change (fold change of POST vs PRE training modality) for chronic training comparisons in men and women. Black dots represent miRNAs that were significantly higher following the specific training and red dots represent miRNAs that were significantly lower following training (*p* ≤ 0.05). The number of up‐ and downregulated miRNAs after training are depicted in each graph (c). Total EV miRNA statistical analysis was performed with a three‐way ANOVA. Differentially expressed miRNAs were identified using edgeR software. *N =* 5. Statistical significance set at *p* = 0.05.

EV miRNA was 184% higher in women than men, independent of any training (main effect of biological sex: *p* = 0.0242). Additionally, chronic training, independent of sex and acute training, elevated EV miRNA content by 29% (main effect of training: *p* = 0.0309; Figure 4A). Although the raw counts changed, the top 25 most enriched miRNAs in EVs remained consistent across the 12‐week RET program and were similar in men and women, as 78% of the top 25 most enriched miRNAs were shared in all four resting conditions (Figure 4B).

A total of eight within sex comparisons were made to identify the impact of acute and chronic RET on EV miRNA contents in men and women. The differentially expressed miRNAs and the number of target mRNAs, as predicted by IPA, for each comparison are detailed in Table S1. Volcano plots depicting miRNA Fold Change and *p*‐value for each chronic training comparison are depicted in Figure 4C.

### 3.4 Targeted pathway analysis of EV miRNAs

**FIGURE 5:**
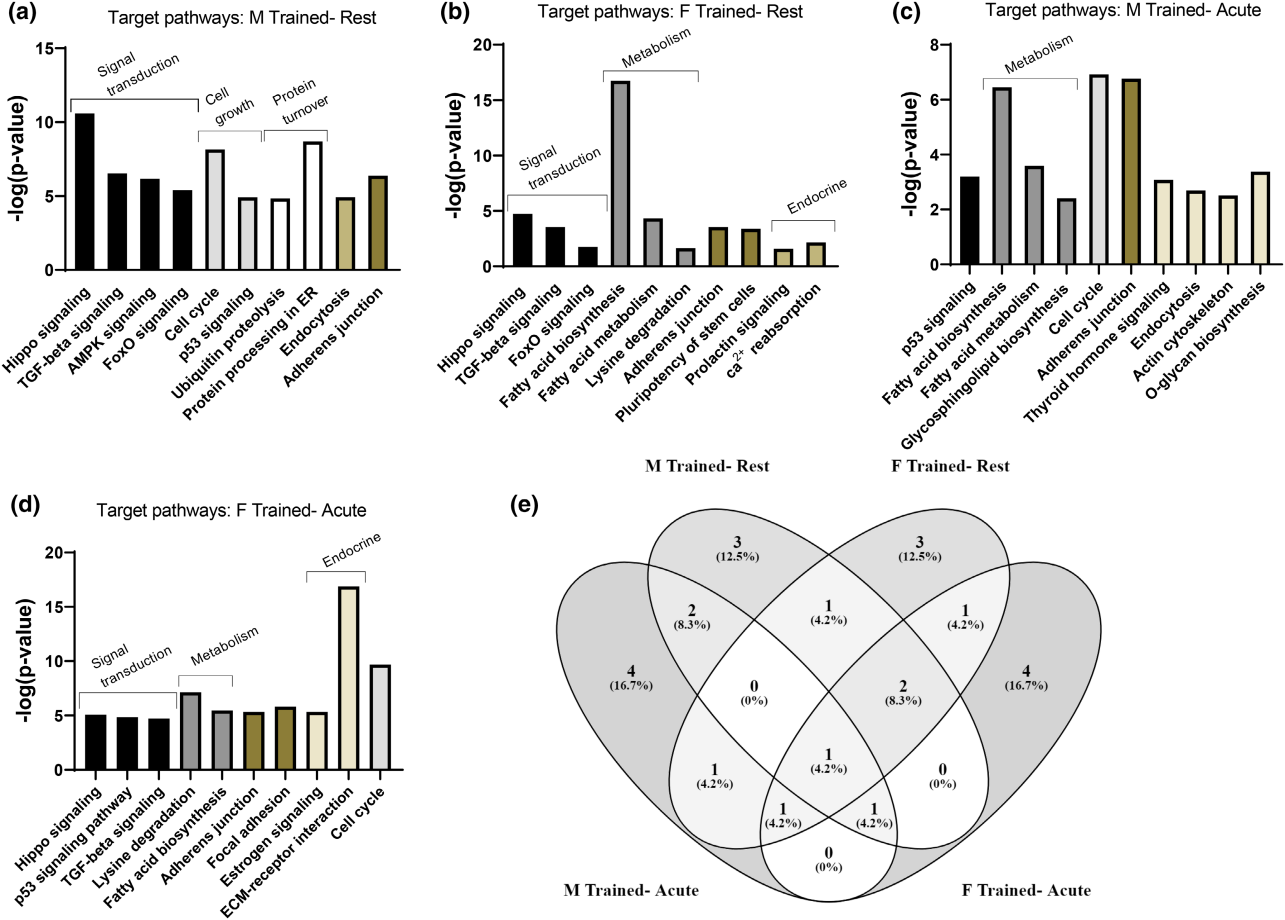
General target pathways of differentially expressed EV miRNAs. The top 10 most significant KEGG pathways containing target genes of the differentially expressed miRNAs for each of the four chronic exercise comparisons in men and women, determined using DIANA miRPath (a–d). Pathways are grouped into general KEGG designations and then sorted by *p*‐value. A Venn diagram depicts the overlapping target pathways between each of the comparisons (e).

**FIGURE 6:**
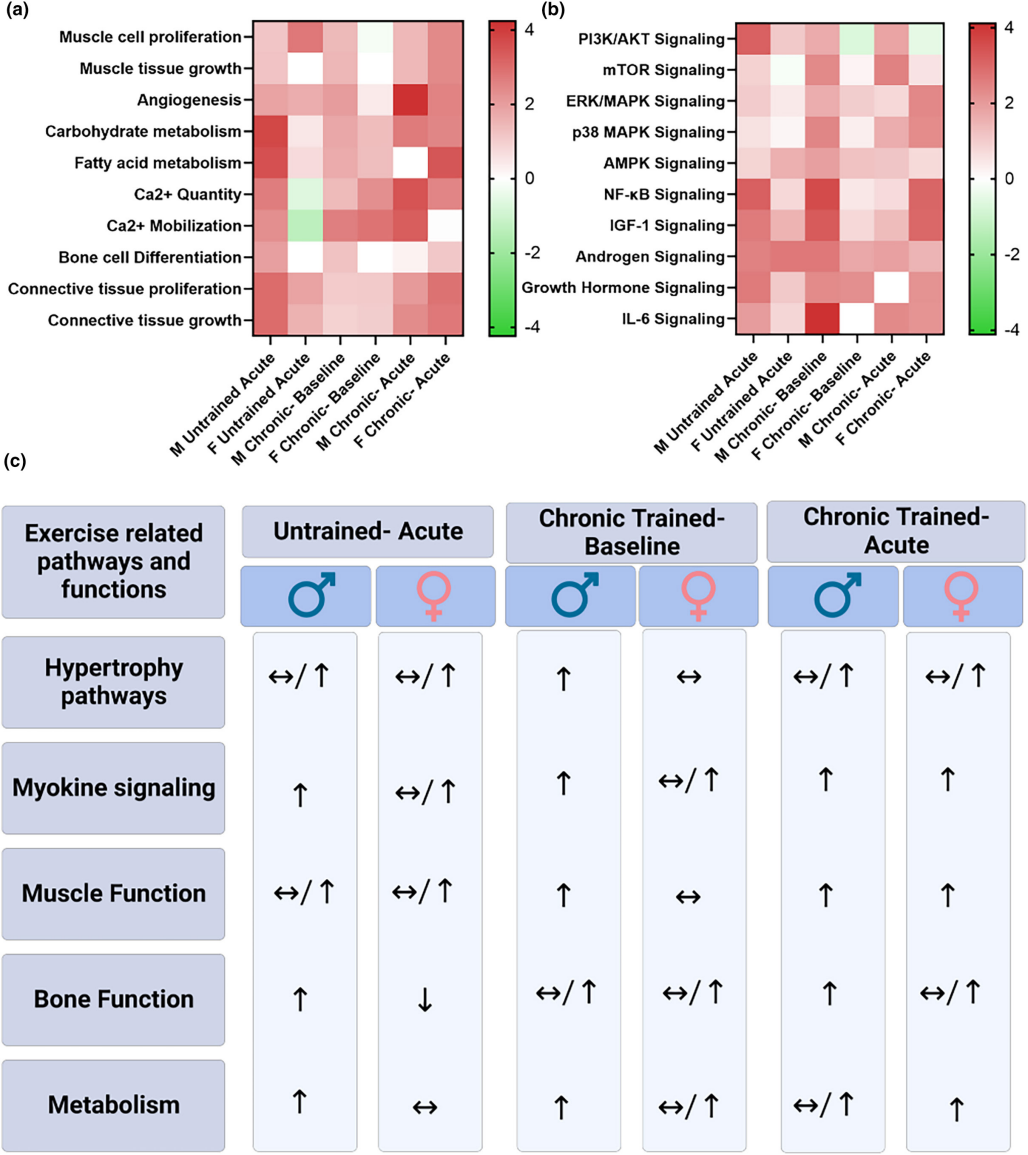
Predicted regulation of exercise‐related pathways by EV miRNAs. Heatmaps were generated in Ingenuity Pathways Analysis depicting the predicted impact (*z*‐score) of EV miRNA changes (post vs pre) on Exercise‐relevant physiological functions (a) and intracellular signaling pathways (b). Red is predicted to be upregulated following chronic training and green is predicted to be downregulated. Depicted comparisons include effects of an acute bout of exercise, 12‐weeks of chronic training, and an acute bout of exercise following chronic training in men and women. A table depicting the average changes of prominent exercise‐related signaling pathways (hypertrophy and myokine) and exercise‐related physiological functions (muscle, bone, and metabolism) were compiled and averaged from the IPA generated heatmaps (c). Arrows pointing up denote significant upregulation, arrows pointing down denote significant downregulation, and sideways arrows depict no significant differences. Functions with multiple arrows had varied responses.

Pathways targeted by the differentially expressed EV miRNAs were generated via two different methods. DIANA miRPath v.3 was used to generate KEGG pathways predicted to be targeted by differentially expressed miRNAs for each comparison (Figure 5a–d). To further probe target pathways and physiological functions, IPA was utilized to generate canonical pathways predicted to be targeted differently for each comparison using the mRNA targets of each miRNA. Enriched canonical pathways for each comparison are detailed in File S3. Additionally, the IPA Functional Analysis feature was utilized to predict the impact that the differentially expressed miRNAs have on various biological functions and diseases for each comparison (File S4). Using IPA generated *Z*‐scores, heatmaps of pathways and biological functions (Figure 6a–c) known to be related to and regulated by RET were generated for acute and chronic exercise training comparisons in men and women. Resistance exercise‐relevant physiological functions and intracellular pathways included those involved with muscle, bone, connective tissue, inflammation, and metabolism.

### 3.5 Predicted EV miRNA Akt pathway regulation reflects intramuscular Akt content

**FIGURE 7:**
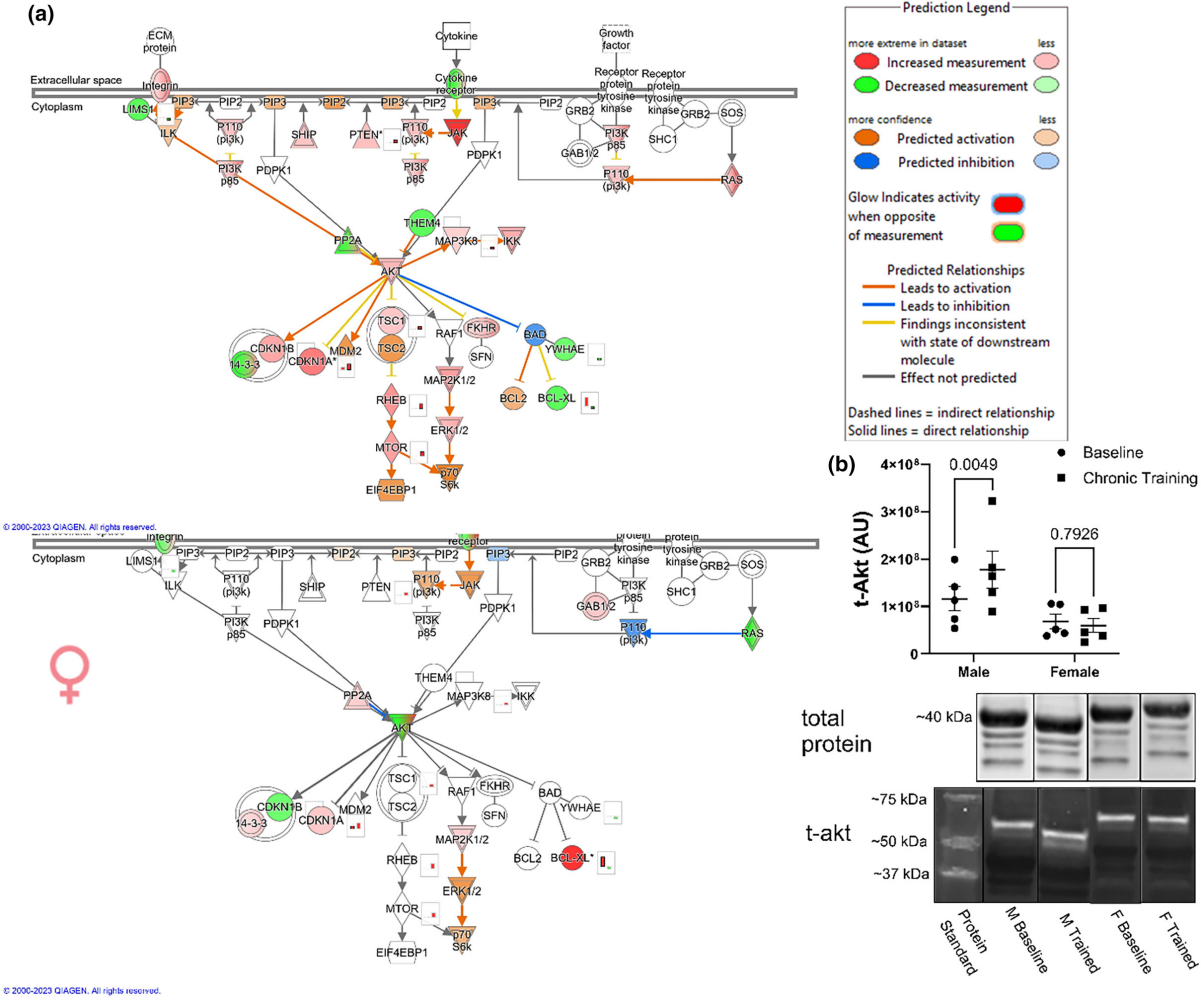
Predicted and measured Akt pathway regulation at rest following chronic resistance training in men and women. IPA generated schematic of the Akt pathway (a) in men (top) and women (bottom). Color coded symbols represent direction of predicted mRNA regulation by differentially expressed EV miRNAs from pre‐ to post‐chronic resistance exercise training and are described in the prediction legend. Total amount of Akt from biopsied skeletal muscle, normalized to total protein, before and after chronic resistance exercise training in resting men and women, respectively, measured via western blotting (b). Representative blot shown, blot single lane images have been vertically spliced to show resting conditions and male–female images side‐by‐side. Complete blot images can be found in supplementary materials. *N* = 5. Akt was analyzed via two‐way ANOVA.

Following exercise training, numerous hypertrophy related pathways were predicted to have been regulated differently in men and women. Concurrent exercise training alters how circulating EV miRNAs are predicted to regulate gene expression of components of Akt pathway more positively in men than women (Figure 7a). In the subset of subjects that had EV miRNA sequencing performed, total intramuscular Akt from biopsied muscle samples (Figure 7b) increased following the 12‐week concurrent training program in men (+57%; *p* = 0.0049) but not women. In these subjects, total lean body mass (Table S3) increased in both men (+2.34 kg; *p* = 0.0022) and women (+1.38 kg; *p* = 0.0157) following the 12 weeks concurrent training program.

## 4 DISCUSSION

Extracellular vesicles continue to emerge as important biomarkers and signaling factors that contribute to exercise adaptations. The present study provides novel descriptive data characterizing the impact of both acute and chronic concurrent resistance and interval exercise training on circulating EV abundance, surface marker expression, and miRNA contents in young men and women. This study fills longstanding gaps in the EV literature as previous reports fall short in determining whether biological sex impacts the EV response to exercise training. Additionally, we provide evidence that circulating EVs contain miRNA contents that support pathways that are supportive of musculoskeletal exercise adaptations, with the added insight that these responses differ between men and women.

### 4.1 Acute exercise elevates circulating EVs after 12 weeks of training in men but not women

It has been well‐established that acute bouts of endurance exercise elevate circulating EV amounts, the majority of which appear to originate from circulatory cells (Brahmer et al., 2019; Estrada et al., 2021; Frühbeis et al., 2015). This increase may be specific to endurance exercise, however, as acute bouts of resistance exercise in untrained individuals don't alter circulating EV amount (Conkright, Beckner, Sterczala, et al., 2022; Gu et al., 2022; Just et al., 2020). The present AHRET results in the untrained men and women are aligned with these previous studies. However, following the 12‐week concurrent exercise training period, the AHRET bout increased circulating EVs in men only, suggesting a sex‐specific adaptation to the training program. Two previous studies observed that 6 and 8 weeks of resistance training did not alter circulating EV amounts at rest; however, neither study tested the acute exercise response in these subjects (Estébanez et al., 2021; Gu et al., 2022). EV release is regulated, at least in part, by origin cell oxidative metabolism (Bebelman et al., 2018; Estrada et al., 2021; Kargl et al., 2023). Importantly, while strength and lean body mass improved during this training program, maximal oxygen consumption did not, suggesting the acute exercise EV increase in trained men was not driven by improved aerobic capacity. The potential explanations for the lack of improvement in aerobic capacity during this concurrent training program have been discussed recently (Sterczala et al., 2024), with the likelihood that the interval intensity was not great enough to elicit strong increases in maximal oxygen consumption despite improvements in endurance performance tests (Sterczala et al., 2023). To interrogate whether the increase in circulating EV abundance following AHRET in trained men was exercise load related we evaluated the absolute and relative intensities of the baseline and 12‐week AHRET bouts. There were no sex or time differences observed for relative intensity of the acute exercise, with each group near the goal of 75% of their 1‐RM over the six sets. As expected, men had a far greater total load lifted across the six sets than women, and training elevated total load lifted by ~26% and 12% in men and women, respectively. Despite the large differences in total load between men and women at baseline and post‐training, total EV abundance was not regulated by sex, only the response to AHRET in trained subjects was.

A potential regulator of EV contents and release that differs between men and women is muscle fiber type composition. Women appear to have a greater proportion of oxidative type I fibers than men (Nuzzo, 2024) and highly oxidative muscle tissue has been found to release more EVs and release EVs with differing miRNA contents than glycolytic tissue (Estrada et al., 2021; Kargl et al., 2023; Nie et al., 2019). This is slightly counter to our findings as only men had an increase in EV abundance following training, but it may have contributed to the miRNA differences. This interpretation of the data should be applied with caution, however, as muscle is a minor contributor (1%–5%; Brahmer et al., 2019; Estrada et al., 2021; Frühbeis et al., 2015; Ismaeel et al., 2023) to the circulating population of EVs at rest and immediately following exercise. The increase in circulating EV abundance in trained men is particularly interesting as it is a somewhat unique response to chronic training for exerkines. Circulating concentrations of many traditional exerkines such as IGF‐1, IL‐6, BDNF, and follistatin are elevated following acute resistance exercise in untrained individuals and either remain elevated in trained individuals or are attenuated following repeated training stimuli (Croft et al., 2009; Nash et al., 2023; Nindl & Pierce, 2010; Zunner et al., 2022). Future work should analyze EV concentrations in women following a longer training period (>6 months) to determine if men develop a training effect more quickly than women or if it is a sex‐based difference.

### 4.2 EV subpopulations sensitive to acute but not chronic training

We utilized imaging flow cytometry to characterize and confirm presence of common markers for exosomes, microvesicles, apoptotic bodies, and muscle‐derived vesicles. Imaging flow cytometry offers superiority over traditional methods for EV research as it allows for the analysis of single EVs in the exosome size range (Tertel et al., 2020). Although we utilized SEC columns that isolate vesicles in the 70–1000 nm size range, the majority of labeled EVs were CD9^+^, indicating a large percentage of small EVs/exosomes. This was consistent with the nanosight sizing data showing peaks in the small EV size range (110 and 160 nms.). Roughly 0.5%–2% of circulating EVs were muscle‐derived (SGCA^+^), which is consistent with a growing number of studies (Annibalini et al., 2019; Brahmer et al., 2019; Estrada et al., 2021; Guescini et al., 2015; Ismaeel et al., 2023) demonstrating that only a small fraction of circulating EVs at rest or following exercise originate from muscle. Since SGCA may not be the most accurate marker for muscle‐derived EVs (Estrada et al., 2021), we also checked for presence of muscle‐derived miRNAs (myomiRs; miRNA‐1, ‐133a and b, ‐206, ‐486), which were present at only very low amounts, confirming low abundance of circulating muscle‐derived EVs. A potential confounding factor of whether muscle‐derived EVs comprise a significant proportion of the circulating EV population after exercise is timing of sample collection. A recent study in mice reported that electrical pulse stimulated muscle contractions did not change EV myomiR amounts immediately following exercise, but 90 min following exercise EV myomiRs increased 10–15 fold (Kawanishi et al., 2023). To limit effects of previous exercise on circulating EVs, a 72‐h washout period was utilized. To better understand circulating EV responses to resistance training, additional timepoints in future studies may need to be evaluated to see if this data is reflected in humans as it would suggest muscle‐derived EVs enter circulation at a delayed rate.

Surface marker proportions for the four measured EV markers largely unaffected by sex or chronic training status. And the acute exercise effects observed are consistent with our previous data demonstrating that AHRET increases microvesicle proportion, independent of sex (Conkright, Beckner, Sterczala, et al., 2022). In that report, SGCA proportion was unaffected, but fluorescent intensity was decreased following AHRET, whereas in the present study we found decreased SGCA proportion following AHRET. In totality, our data suggests that the internal contents of EVs were more sensitive to chronic training induced changes than the EV surface markers that we measured. A combination of these two approaches, examining the contents of different subpopulations of EVs, especially exosomes, following resistance exercise, could be a rich avenue for future work.

### 4.3 Concurrent training differentially alters EV miRNA patterns in men and women

Biological sex impacts circulating EV characteristics at rest and following acute bouts of resistance training (Beckner et al., 2022; Conkright, Beckner, Sterczala, et al., 2022), but until this study, the impact of a long‐term concurrent resistance and interval training program on EV content characteristics in men and women was unexplored. Circulating EVs from women contained overall greater amounts of miRNA compared to men, independent of acute or chronic training status. Examination of the most expressed miRNAs across comparisons reveals numerous miRNAs that are highly expressed in EVs, regardless of training status or biological sex, as 18 of the top 25 most enriched miRNAs were shared between all groups. While the most enriched miRNAs were similar in men and women, the expression responses of miRNAs to acute and chronic training were vastly different. In men, when examining resting miRNA contents of EVs, 31 miRNAs were lower and 8 were higher after 12 weeks of chronic training compared to baseline, while women saw much less impact of chronic training on resting miRNA contents, with 7 down and 5 upregulated miRNAs. This pattern was inverted when examining the effects of chronic training on the response to the AHRET bout as only 7 miRNAs were differentially expressed in men when comparing post‐AHRET miRNAs at baseline and following 12 weeks of training compared to 32 miRNAs differentially expressed in women. This suggests that, following the 12‐week training period, miRNA contents changed more in men compared to women at rest. However, following the AHRET bout EV miRNA contents in women were more responsive to acute exercise after the 12‐week training compared to men. In totality this shows that AHRET impacts individual EV miRNA expression more in untrained than trained men, whereas the effect is opposite in women. It appears then that EV contents, like other exerkines, display some sex‐based responses to acute and chronic training. Immediately following RET, circulating concentration of testosterone, cortisol, and IL‐6 are greater in men than women (Benini et al., 2015) and the most abundant isoform of IGF‐1 increases in men but not women (Pierce et al., 2020). Additionally, Pierce and colleagues observed that, while GH concentration increased in untrained men and women following the same protocol of AHRET performed in the current report, increases were sustained longer in men (Pierce et al., 2020). Interestingly, a follow‐up study done in resistance‐trained individuals found that the AHRET bout induced similar increases in GH in men and women, but that only women experienced exercise‐induced increases in IGF‐1 isoforms, with concentration greater than men (Hatfield et al., 2021). The miRNA data from the current report follows this pattern, as the 12‐week concurrent training period induced larger changes in EV miRNA contents in women compared to men. Given these data, it is possible that the circulating anabolic signaling environment in untrained men is more robust than in women and is normalized or even higher in woman following long‐term exercise training.

To further explore this idea, we utilized pathway analysis software to determine if the differentially expressed miRNAs were predicted to target pathways related to exercise training adaptations. Many of the general pathways predicted to be most heavily targeted by the differentially expressed EV miRNAs in men and women following chronic exercise were signal transduction and metabolism pathways. Using IPA, we identified specific canonical pathways and cellular functions related to resistance exercise and predicted the directionality of regulation based on the differentially expressed miRNAs for each exercise comparison. The initial acute exercise stimuli in untrained subjects altered EV miRNA contents in men and women, with overall positive effects predicted for numerous exercise‐relevant pathways and functions. The effects were more pronounced in men when it came to functions and pathways related to bone homeostasis, metabolism, and myokine (GH, IGF‐1, and IL‐6) signaling. This supports the previously discussed work highlighting the differences in myokine signaling between men and women following acute RT (Benini et al., 2015; Pierce et al., 2020). While it is unknown if the circulating EV miRNA changes immediately following AHRET in untrained individuals have any influence on circulating myokine concentration, it does show that the circulating milieu in untrained men following AHRET is consistent and supports elevated myokine signaling.

While, the 12‐week training program altered resting EV miRNA contents in manners predicted to positively regulate resistance exercise‐related adaptations in both sexes, the effects were more pronounced in men compared to women. Additionally, the predicted regulation of the Akt pathway, a central upstream regulator of mTOR and hypertrophy (Bodine et al., 2001; Glass, 2010), was much more robust in men than women, and this was reflected by increased content of skeletal muscle total‐Akt protein in men, but not women, following the training program. While the descriptive nature of this report precludes us from determining if EV miRNAs contributed to this sex difference, circulating EVs can be taken in by skeletal muscle (Aswad et al., 2014; Sahu et al., 2021). Additionally, since miRNAs primarily act to degrade messenger RNAs (O'Brien et al., 2018), effects would more likely be seen on the total protein level as opposed to the phosphorylation level, which was not different between men and women at any timepoint (data not shown) in the present study. However, following AHRET the predicted miRNA regulatory response for resistance exercise‐related pathways and functions in trained individuals was almost identical in men and women. Longer training studies will have to be completed to determine if the impacts of chronic training on predicted pathway regulation of circulating EV miRNAs is merely delayed in women compared to men during relatively low‐volume training programs.

### 4.4 Limitations

While this study provides evidence for our conclusions, it is not without limitations. The study design lacked a non‐intervention control, with participant baseline visits serving as the control. Participant nutritional intake was not tracked or controlled for. Since participants gained weight following the training program despite the presence of the exercise program, it is likely that a slight dietary change occurred during the intervention that could have potentially influenced how EVs adapted to training. An unfortunate result of the study design was that the concurrent training program elicited strength increases but not increased VO_2_max, limiting our ability to determine how an exercise program that improves strength and aerobic capacity impacts circulating EVs. Additionally, we utilized SEC columns with a size range of 70–1000 nm for our EV isolations. Since apoptotic bodies can range from 50 to 5000 nm (Kakarla et al., 2020), it is possible we did not capture the entire population and may have underestimated their proportions across all groups. Lastly, our content identification was limited to EV miRNAs and only examined contents immediately following exercise. EVs are well known carriers of other RNA subtypes as well as lipids and protein. Our study provides a strong framework demonstrating that EV contents are sensitive to miRNA changes in a sex‐dependent manner; therefore, a multi‐omic approach may be warranted to determine if other EV macromolecule contents are regulated similarly.

### 4.5 Conclusion

The current study represents the first reported comparison of circulating EV characteristics and miRNA contents between men and women following chronic concurrent exercise training at rest and following acute resistance exercise. Sex‐based differences in exercise responses are well characterized and regulated by genetic and hormonal mechanisms. We observed sex‐specific responses to chronic concurrent exercise training highlighted by an increase in circulating EV number following AHRET in trained men but not women and differing predicted regulation of several pathways and functions key for adaptations to RET. This study represents a first step in identifying sexually dimorphic EV responses to concurrent exercise training and acute resistance training. Future studies should explore whether EVs have mechanistic roles in the adaptive response to exercise training in men and women, as well as identify sexually dimorphic surface markers, non‐miRNA contents and the cellular origins of post‐exercise EVs.

## AUTHOR CONTRIBUTIONS

CK and BN conceived and designed research. CK, AS, KK, DS, and BM collected data and performed experiments. CK analyzed data, interpreted results of experiments, prepared tables/figures and drafted manuscript. CK, AS, DS, WC MH, KK, BM, JG, TO, SW, AS, FA, BN edited and revised manuscript. CK, AS, DS, WC MH, KK, BM, JG, TO, SW, AS, FA, BN approved final version of manuscript.

## FUNDING INFORMATION

This work was supported by United Kingdom Ministry of Defense, award no. WGCC 5.5.6—Task 0107.

## CONFLICT OF INTEREST STATEMENT

No conflicts of interest, financial or otherwise, are declared by the authors.

## ETHICS STATEMENT

This study was approved by the University of Pittsburgh Institutional Review Board (IRB #19030387) and United Kingdom Ministry of Defense Research Ethics Committee (REF: 903/MODREC/18) in accordance with the Declaration of Helsinki.

## DISCLAIMERS

The results of the present study do not constitute endorsement of the product by the authors, the Department of Defense, or the U.S. Government. Citations of commercial organizations and trade names in this report do not constitute an official Department of Defense endorsement or approval of the products or services of these organizations. The results of the study are presented clearly, honestly, and without fabrication, falsification, or inappropriate data manipulation.

## Supporting information


Table S1.
Table S2.
Table S3.

Data S1.

File S1.

File S2.

File S3.

File S4.

## Data Availability

The data that support the findings of this study will be made available upon reasonable request from the corresponding author. All requests must also have an approved data use agreement signed and approved by the University of Pittsburgh. Supplemental materials are available through FigShare (https://figshare.com/s/47d3e50be0bb11132714) Raw and processed miRNA sequencing data can be accessed on the NIH Gene Expression Omnibus database, GEO Accession #GSE232700.

## References

[phy216016-bib-0001] Abdelsaid, K. , Sudhahar, V. , Harris, R. A. , Das, A. , Youn, S.‐W. , Liu, Y. , McMenamin, M. , Hou, Y. , Fulton, D. , Hamrick, M. W. , Tang, Y. , Fukai, T. , & Ushio‐Fukai, M. (2022). Exercise improves angiogenic function of circulating exosomes in type 2 diabetes: Role of exosomal SOD3. The FASEB Journal, 36(3), e22177. 10.1096/fj.202101323R 35142393 PMC8880294

[phy216016-bib-0002] Akers, J. C. , Gonda, D. , Kim, R. , Carter, B. S. , & Chen, C. C. (2013). Biogenesis of extracellular vesicles (EV): Exosomes, microvesicles, retrovirus‐like vesicles, and apoptotic bodies. Journal of Neuro‐Oncology, 113(1), 1–11. 10.1007/s11060-013-1084-8 23456661 PMC5533094

[phy216016-bib-0003] Annibalini, G. , Contarelli, S. , Lucertini, F. , Guescini, M. , Maggio, S. , Ceccaroli, P. , Gervasi, M. , Ferri Marini, C. , Fardetti, F. , Grassi, E. , Stocchi, V. , Barbieri, E. , & Benelli, P. (2019). Muscle and systemic molecular responses to a single flywheel based iso‐inertial training session in resistance‐trained men. Frontiers in Physiology, 10, 554. 10.3389/fphys.2019.00554 31143128 PMC6521220

[phy216016-bib-0004] Aswad, H. , Forterre, A. , Wiklander, O. P. B. , Vial, G. , Danty‐Berger, E. , Jalabert, A. , Lamazière, A. , Meugnier, E. , Pesenti, S. , Ott, C. , Chikh, K. , El‐Andaloussi, S. , Vidal, H. , Lefai, E. , Rieusset, J. , & Rome, S. (2014). Exosomes participate in the alteration of muscle homeostasis during lipid‐induced insulin resistance in mice. Diabetologia, 57(10), 2155–2164. 10.1007/s00125-014-3337-2 25073444 PMC4153976

[phy216016-bib-0005] Barcellos, N. , Cechinel, L. R. , de Meireles, L. C. F. , Lovatel, G. A. , Bruch, G. E. , Carregal, V. M. , Massensini, A. R. , Dalla Costa, T. , Pereira, L. O. , & Siqueira, I. R. (2020). Effects of exercise modalities on BDNF and IL‐1β content in circulating total extracellular vesicles and particles obtained from aged rats. Experimental Gerontology, 142, 111124. 10.1016/j.exger.2020.111124 33148515

[phy216016-bib-0006] Bartel, D. P. (2009). MicroRNAs: Target recognition and regulatory functions. Cell, 136(2), 215–233. 10.1016/j.cell.2009.01.002 19167326 PMC3794896

[phy216016-bib-0007] Bebelman, M. P. , Smit, M. J. , Pegtel, D. M. , & Baglio, S. R. (2018). Biogenesis and function of extracellular vesicles in cancer. Pharmacology & Therapeutics, 188, 1–11. 10.1016/j.pharmthera.2018.02.013 29476772

[phy216016-bib-0008] Beckner, M. E. , Main, L. , Tait, J. L. , Martin, B. J. , Conkright, W. R. , & Nindl, B. C. (2022). Circulating biomarkers associated with performance and resilience during military operational stress. European Journal of Sport Science, 22(1), 72–86. 10.1080/17461391.2021.1962983 34346851

[phy216016-bib-0009] Benini, R. , Prado Nunes, P. R. , Orsatti, C. L. , Barcelos, L. C. , & Orsatti, F. L. (2015). Effects of acute total body resistance exercise on hormonal and cytokines changes in men and women. The Journal of Sports Medicine and Physical Fitness, 55(4), 337–344. 25853878

[phy216016-bib-0010] Bodine, S. C. , Stitt, T. N. , Gonzalez, M. , Kline, W. O. , Stover, G. L. , Bauerlein, R. , Zlotchenko, E. , Scrimgeour, A. , Lawrence, J. C. , Glass, D. J. , & Yancopoulos, G. D. (2001). Akt/mTOR pathway is a crucial regulator of skeletal muscle hypertrophy and can prevent muscle atrophy in vivo. Nature Cell Biology, 3(11), Article 11. 10.1038/ncb1101-1014 11715023

[phy216016-bib-0011] Brahmer, A. , Neuberger, E. , Esch‐Heisser, L. , Haller, N. , Jorgensen, M. M. , Baek, R. , Möbius, W. , Simon, P. , & Krämer‐Albers, E.‐M. (2019). Platelets, endothelial cells and leukocytes contribute to the exercise‐triggered release of extracellular vesicles into the circulation. Journal of Extracellular Vesicles, 8(1), 1615820. 10.1080/20013078.2019.1615820 31191831 PMC6542154

[phy216016-bib-0012] Chow, L. S. , Gerszten, R. E. , Taylor, J. M. , Pedersen, B. K. , van Praag, H. , Trappe, S. , Febbraio, M. A. , Galis, Z. S. , Gao, Y. , Haus, J. M. , Lanza, I. R. , Lavie, C. J. , Lee, C.‐H. , Lucia, A. , Moro, C. , Pandey, A. , Robbins, J. M. , Stanford, K. I. , Thackray, A. E. , … Snyder, M. P. (2022). Exerkines in health, resilience and disease. Nature Reviews Endocrinology, 18(5), 273–289. 10.1038/s41574-022-00641-2 PMC9554896 35304603

[phy216016-bib-0013] Colombo, M. , Raposo, G. , & Théry, C. (2014). Biogenesis, secretion, and intercellular interactions of exosomes and other extracellular vesicles. Annual Review of Cell and Developmental Biology, 30(1), 255–289. 10.1146/annurev-cellbio-101512-122326 25288114

[phy216016-bib-0014] Conkright, W. R. , Beckner, M. E. , Sahu, A. , Mi, Q. , Clemens, Z. J. , Lovalekar, M. , Flanagan, S. D. , Martin, B. J. , Ferrarelli, F. , Ambrosio, F. , & Nindl, B. C. (2022). Men and women display distinct extracellular vesicle biomarker signatures in response to military operational stress. Journal of Applied Physiology, 132(5), 1125–1136. 10.1152/japplphysiol.00664.2021 35297690 PMC9054257

[phy216016-bib-0015] Conkright, W. R. , Beckner, M. E. , Sterczala, A. J. , Mi, Q. , Lovalekar, M. , Sahu, A. , Krajewski, K. T. , Martin, B. J. , Flanagan, S. D. , Greeves, J. P. , O'Leary, T. J. , Wardle, S. L. , Ambrosio, F. , & Nindl, B. C. (2022). Resistance exercise differentially alters extracellular vesicle size and subpopulation characteristics in healthy men and women: An observational cohort study. Physiological Genomics, 54(9), 350–359. 10.1152/physiolgenomics.00171.2021 35816651

[phy216016-bib-0016] Croft, L. , Bartlett, J. D. , MacLaren, D. P. M. , Reilly, T. , Evans, L. , Mattey, D. L. , Nixon, N. B. , Drust, B. , & Morton, J. P. (2009). High‐intensity interval training attenuates the exercise‐induced increase in plasma IL‐6 in response to acute exercise. Applied Physiology, Nutrition, and Metabolism = Physiologie Appliquee, Nutrition et Metabolisme, 34(6), 1098–1107. 10.1139/H09-117 20029520

[phy216016-bib-0017] Estébanez, B. , Visavadiya, N. P. , de Paz, J. A. , Whitehurst, M. , Cuevas, M. J. , González‐Gallego, J. , & Huang, C.‐J. (2021). Resistance training diminishes the expression of exosome CD63 protein without modification of plasma miR‐146a‐5p and cfDNA in the elderly. Nutrients, 13(2), 665. 10.3390/nu13020665 33669497 PMC7922765

[phy216016-bib-0018] Estrada, A. L. , Valenti, Z. J. , Hehn, G. , Amorese, A. J. , Williams, N. S. , Balestrieri, N. P. , Deighan, C. , Allen, C. P. , Spangenburg, E. E. , Kruh‐Garcia, N. A. , & Lark, D. S. (2021). Extracellular vesicle secretion is tissue‐dependent ex vivo and skeletal muscle myofiber extracellular vesicles reach the circulation in vivo. American Journal of Physiology. Cell Physiology, 322, C246–C259. 10.1152/ajpcell.00580.2020 34910603 PMC8816621

[phy216016-bib-0019] Frühbeis, C. , Helmig, S. , Tug, S. , Simon, P. , & Krämer‐Albers, E.‐M. (2015). Physical exercise induces rapid release of small extracellular vesicles into the circulation. Journal of Extracellular Vesicles, 4(1), 28239. 10.3402/jev.v4.28239 26142461 PMC4491306

[phy216016-bib-0020] Gao, L. , Wang, H.‐J. , Tian, C. , & Zucker, I. H. (2021). Skeletal muscle Nrf2 contributes to exercise‐evoked systemic antioxidant defense via extracellular vesicular communication. Exercise and Sport Sciences Reviews, 49(3), 213–222. 10.1249/JES.0000000000000257 33927165 PMC8195856

[phy216016-bib-0021] Garner, R. T. , Weiss, J. A. , Nie, Y. , Sullivan, B. P. , Kargl, C. K. , Drohan, C. J. , Kuang, S. , Stout, J. , & Gavin, T. P. (2022). Effects of obesity and acute resistance exercise on skeletal muscle angiogenic communication pathways. Experimental Physiology, 107, 906–918. 10.1113/EP090152 35561231

[phy216016-bib-0022] Glass, D. J. (2010). PI3 kinase regulation of skeletal muscle hypertrophy and atrophy. Current Topics in Microbiology and Immunology, 346, 267–278. 10.1007/82_2010_78 20593312

[phy216016-bib-0023] Greeves, J. P. , Beck, B. , Nindl, B. C. , & O'Leary, T. J. (2023). Current risks factors and emerging biomarkers for bone stress injuries in military personnel. Journal of Science and Medicine in Sport, 26(Suppl 1), S14–S21. 10.1016/j.jsams.2023.04.006 37188615

[phy216016-bib-0024] Gu, T. , Just, J. , Stenz, K. T. , Yan, Y. , Sieljacks, P. , Wang, J. , Groennebaek, T. S. , Jakobsgaard, J. E. , Rindom, E. , Herskind, J. , Gravholt, A. , Lassen, T. R. , Jørgensen, M. , Bæk, R. , Gutiérrez‐Jiménez, E. , Iversen, N. K. , Rasmussen, P. M. , Nyengaard, J. R. , Jørgensen, M. M. , … Drasbek, K. R. (2022). The role of plasma extracellular vesicles in remote ischemic conditioning and exercise‐induced ischemic tolerance. International Journal of Molecular Sciences, 23(6), 3334. 10.3390/ijms23063334 35328755 PMC8951333

[phy216016-bib-0025] Guescini, M. , Canonico, B. , Lucertini, F. , Maggio, S. , Annibalini, G. , Barbieri, E. , Luchetti, F. , Papa, S. , & Stocchi, V. (2015). Muscle releases alpha‐Sarcoglycan positive extracellular vesicles carrying miRNAs in the bloodstream. PLoS One, 10(5), e0125094. 10.1371/journal.pone.0125094 25955720 PMC4425492

[phy216016-bib-0026] Hatfield, D. L. , Kraemer, W. J. , Volek, J. S. , Nindl, B. C. , Caldwell, L. K. , Vingren, J. L. , Newton, R. U. , Häkkinen, K. , Lee, E. C. , Maresh, C. M. , & Hymer, W. C. (2021). Hormonal stress responses of growth hormone and insulin‐like growth factor‐I in highly resistance trained women and men. Growth Hormone & IGF Research, 59, 101407. 10.1016/j.ghir.2021.101407 34118743

[phy216016-bib-0027] Hsu, S.‐D. , Lin, F.‐M. , Wu, W.‐Y. , Liang, C. , Huang, W.‐C. , Chan, W.‐L. , Tsai, W.‐T. , Chen, G.‐Z. , Lee, C.‐J. , Chiu, C.‐M. , Chien, C.‐H. , Wu, M.‐C. , Huang, C.‐Y. , Tsou, A.‐P. , & Huang, H.‐D. (2011). miRTarBase: A database curates experimentally validated microRNA–target interactions. Nucleic Acids Research, 39, D163–D169. 10.1093/nar/gkq1107 21071411 PMC3013699

[phy216016-bib-0028] Ismaeel, A. , Van Pelt, D. W. , Hettinger, Z. R. , Fu, X. , Richards, C. I. , Butterfield, T. A. , Petrocelli, J. J. , Vechetti, I. J. , Confides, A. L. , Drummond, M. J. , & Dupont‐Versteegden, E. E. (2023). Extracellular vesicle distribution and localization in skeletal muscle at rest and following disuse atrophy. Skeletal Muscle, 13(1), 6. 10.1186/s13395-023-00315-1 36895061 PMC9999658

[phy216016-bib-0029] Just, J. , Yan, Y. , Farup, J. , Sieljacks, P. , Sloth, M. , Venø, M. , Gu, T. , de Paoli, F. V. , Nyengaard, J. R. , Bæk, R. , Jørgensen, M. M. , Kjems, J. , Vissing, K. , & Drasbek, K. R. (2020). Blood flow‐restricted resistance exercise alters the surface profile, miRNA cargo and functional impact of circulating extracellular vesicles. Scientific Reports, 10, 5835. 10.1038/s41598-020-62456-3 32245988 PMC7125173

[phy216016-bib-0030] Kakarla, R. , Hur, J. , Kim, Y. J. , Kim, J. , & Chwae, Y.‐J. (2020). Apoptotic cell‐derived exosomes: Messages from dying cells. Experimental & Molecular Medicine, 52(1) Article 1, 1–6. 10.1038/s12276-019-0362-8 31915368 PMC7000698

[phy216016-bib-0031] Kargl, C. K. , Jia, Z. , Shera, D. A. , Sullivan, B. P. , Burton, L. C. , Kim, K. H. , Nie, Y. , Hubal, M. J. , Shannahan, J. H. , Kuang, S. , & Gavin, T. P. (2023). Angiogenic potential of skeletal muscle derived extracellular vesicles differs between oxidative and glycolytic muscle tissue in mice. Scientific Reports, 13(1) Article 1, 18943. 10.1038/s41598-023-45787-9 37919323 PMC10622454

[phy216016-bib-0032] Karvinen, S. , Korhonen, T.‐M. , Sievänen, T. , Karppinen, J. E. , Juppi, H.‐K. , Jakoaho, V. , Kujala, U. M. , Laukkanen, J. A. , Lehti, M. , & Laakkonen, E. K. (2023). Extracellular vesicles and high‐density lipoproteins: Exercise and oestrogen‐responsive small RNA carriers. Journal of Extracellular Vesicles, 12(2), e12308. 10.1002/jev2.12308 36739598 PMC9899444

[phy216016-bib-0033] Kawanishi, N. , Tominaga, T. , & Suzuki, K. (2023). Electrical pulse stimulation‐induced muscle contraction alters the microRNA and mRNA profiles of circulating extracellular vesicles in mice. American Journal of Physiology. Regulatory, Integrative and Comparative Physiology, 324, R761–R771. 10.1152/ajpregu.00121.2022 37092746

[phy216016-bib-0034] Nash, D. , Hughes, M. G. , Butcher, L. , Aicheler, R. , Smith, P. , Cullen, T. , & Webb, R. (2023). IL‐6 signaling in acute exercise and chronic training: Potential consequences for health and athletic performance. Scandinavian Journal of Medicine & Science in Sports, 33(1), 4–19. 10.1111/sms.14241 36168944 PMC10092579

[phy216016-bib-0035] Nederveen, J. P. , Warnier, G. , Di Carlo, A. , Nilsson, M. I. , & Tarnopolsky, M. A. (2020). Extracellular vesicles and exosomes: Insights from exercise science. Frontiers in Physiology, 11, 604274. 10.3389/fphys.2020.604274 33597890 PMC7882633

[phy216016-bib-0036] Nie, Y. , Sato, Y. , Garner, R. T. , Kargl, C. , Wang, C. , Kuang, S. , Gilpin, C. J. , & Gavin, T. P. (2019). Skeletal muscle‐derived exosomes regulate endothelial cell functions via reactive oxygen species‐activated nuclear factor‐κB signalling. Experimental Physiology, 104, 1262–1273. 10.1113/EP087396 31115069

[phy216016-bib-0037] Nindl, B. C. , Kraemer, W. J. , Gotshalk, L. A. , Marx, J. O. , Volek, J. S. , Bush, F. A. , Häkkinen, K. , Newton, R. U. , & Fleck, S. J. (2001). Testosterone responses after resistance exercise in women: Influence of regional fat distribution. International Journal of Sport Nutrition and Exercise Metabolism, 11(4), 451–465. 10.1123/ijsnem.11.4.451 11915780

[phy216016-bib-0038] Nindl, B. C. , & Pierce, J. R. (2010). Insulin‐like growth factor I as a biomarker of health, fitness, and training status. Medicine and Science in Sports and Exercise, 42(1), 39–49. 10.1249/MSS.0b013e3181b07c4d 20010131

[phy216016-bib-0039] Nuzzo, J. L. (2023). Narrative review of sex differences in muscle strength, endurance, activation, size, fiber type, and strength training participation rates, preferences, motivations, injuries, and neuromuscular adaptations. The Journal of Strength & Conditioning Research, 37(2), 494–536. 10.1519/JSC.0000000000004329 36696264

[phy216016-bib-0040] Nuzzo, J. L. (2024). Sex differences in skeletal muscle fiber types: A meta‐analysis. Clinical Anatomy, 37(1), 81–91. 10.1002/ca.24091 37424380

[phy216016-bib-0041] O'Brien, J. , Hayder, H. , Zayed, Y. , & Peng, C. (2018). Overview of microRNA biogenesis, mechanisms of actions, and circulation. Frontiers in Endocrinology, 9, 402. 10.3389/fendo.2018.00402 30123182 PMC6085463

[phy216016-bib-0042] Pierce, J. R. , Martin, B. J. , Rarick, K. R. , Alemany, J. A. , Staab, J. S. , Kraemer, W. J. , Hymer, W. C. , & Nindl, B. C. (2020). Growth hormone and insulin‐like growth factor‐I molecular weight isoform responses to resistance exercise are sex‐dependent. Frontiers in Endocrinology, 11, 571. 10.3389/fendo.2020.00571 32973684 PMC7472848

[phy216016-bib-0043] Roberts, B. M. , Nuckols, G. , & Krieger, J. W. (2020). Sex differences in resistance training: A systematic review and meta‐analysis. The Journal of Strength & Conditioning Research, 34(5), 1448–1460. 10.1519/JSC.0000000000003521 32218059

[phy216016-bib-0044] Roberts, M. D. , McCarthy, J. J. , Hornberger, T. A. , Phillips, S. M. , Mackey, A. L. , Nader, G. A. , Boppart, M. D. , Kavazis, A. N. , Reidy, P. T. , Ogasawara, R. , Libardi, C. A. , Ugrinowitsch, C. , Booth, F. W. , & Esser, K. A. (2023). Mechanisms of mechanical overload‐induced skeletal muscle hypertrophy: Current understanding and future directions. Physiological Reviews, 103, 2679–2757. 10.1152/physrev.00039.2022 37382939 PMC10625844

[phy216016-bib-0045] Robinson, M. D. , McCarthy, D. J. , & Smyth, G. K. (2010). edgeR: A bioconductor package for differential expression analysis of digital gene expression data. Bioinformatics (Oxford, England), 26(1), 139–140. 10.1093/bioinformatics/btp616 19910308 PMC2796818

[phy216016-bib-0046] Sahu, A. , Clemens, Z. J. , Shinde, S. N. , Sivakumar, S. , Pius, A. , Bhatia, A. , Picciolini, S. , Carlomagno, C. , Gualerzi, A. , Bedoni, M. , Van Houten, B. , Lovalekar, M. , Fitz, N. F. , Lefterov, I. , Barchowsky, A. , Koldamova, R. , & Ambrosio, F. (2021). Regulation of aged skeletal muscle regeneration by circulating extracellular vesicles. Nature Aging, 1–14, 1148–1161. 10.1038/s43587-021-00143-2 PMC9165723 35665306

[phy216016-bib-0047] Sterczala, A. J. , Krajewski, K. T. , Peterson, P. A. , Sekel, N. M. , Lovalekar, M. , Wardle, S. L. , O'Leary, T. J. , Greeves, J. P. , Flanagan, S. D. , Connaboy, C. , & Nindl, B. C. (2023). Twelve weeks of concurrent resistance and interval training improves military occupational task performance in men and women. European Journal of Sport Science, 1–14, 2411–2424. 10.1080/17461391.2023.2239752 37517090

[phy216016-bib-0048] Sterczala, A. J. , Rodriguez‐Ortiz, N. , Feigel, E. D. , Krajewski, K. T. , Martin, B. J. , Sekel, N. M. , Lovalekar, M. , Kargl, C. K. , Koltun, K. J. , Van Eck, C. , Flanagan, S. D. , Connaboy, C. , Wardle, S. L. , O'Leary, T. J. , Greeves, J. P. , & Nindl, B. C. (2024). Skeletal muscle adaptations to high‐intensity, low‐volume concurrent resistance and interval training in recreationally active men and women. Physiological Reports, 12(6), e15953. 10.14814/phy2.15953 38490811 PMC10942853

[phy216016-bib-0049] Sullivan, B. P. , Nie, Y. , Evans, S. , Kargl, C. K. , Hettinger, Z. R. , Garner, R. T. , Hubal, M. J. , Kuang, S. , Stout, J. , & Gavin, T. P. (2022). Obesity and exercise training alter inflammatory pathway skeletal muscle small extracellular vesicle microRNAs. Experimental Physiology, 107(5), 462–475. 10.1113/EP090062 35293040 PMC9323446

[phy216016-bib-0050] Tertel, T. , Görgens, A. , & Giebel, B. (2020). Chapter four—Analysis of individual extracellular vesicles by imaging flow cytometry. In S. Spada & L. Galluzzi (Eds.), Methods in enzymology (Vol. 645, pp. 55–78). Academic Press. 10.1016/bs.mie.2020.05.013 33565978

[phy216016-bib-0051] Théry, C. , Witwer, K. W. , Aikawa, E. , Alcaraz, M. J. , Anderson, J. D. , Andriantsitohaina, R. , Antoniou, A. , Arab, T. , Archer, F. , Atkin‐Smith, G. K. , Ayre, D. C. , Bach, J.‐M. , Bachurski, D. , Baharvand, H. , Balaj, L. , Baldacchino, S. , Bauer, N. N. , Baxter, A. A. , Bebawy, M. , … Zuba‐Surma, E. K. (2018). Minimal information for studies of extracellular vesicles 2018 (MISEV2018): A position statement of the International Society for Extracellular Vesicles and update of the MISEV2014 guidelines. Journal of Extracellular Vesicles, 7(1), 1535750. 10.1080/20013078.2018.1535750 30637094 PMC6322352

[phy216016-bib-0052] Vechetti, I. J. , Peck, B. D. , Wen, Y. , Walton, R. G. , Valentino, T. R. , Alimov, A. P. , Dungan, C. M. , Pelt, D. W. V. , von Walden, F. , Alkner, B. , Peterson, C. A. , & McCarthy, J. J. (2021). Mechanical overload‐induced muscle‐derived extracellular vesicles promote adipose tissue lipolysis. The FASEB Journal, 35(6), e21644. 10.1096/fj.202100242R 34033143 PMC8607211

[phy216016-bib-0053] Whitham, M. , Parker, B. L. , Friedrichsen, M. , Hingst, J. R. , Hjorth, M. , Hughes, W. E. , Egan, C. L. , Cron, L. , Watt, K. I. , Kuchel, R. P. , Jayasooriah, N. , Estevez, E. , Petzold, T. , Suter, C. M. , Gregorevic, P. , Kiens, B. , Richter, E. A. , James, D. E. , Wojtaszewski, J. F. P. , & Febbraio, M. A. (2018). Extracellular vesicles provide a means for tissue crosstalk during exercise. Cell Metabolism, 27(1), 237–251.e4. 10.1016/j.cmet.2017.12.001 29320704

[phy216016-bib-0054] Zunner, B. E. M. , Wachsmuth, N. B. , Eckstein, M. L. , Scherl, L. , Schierbauer, J. R. , Haupt, S. , Stumpf, C. , Reusch, L. , & Moser, O. (2022). Myokines and resistance training: A narrative review. International Journal of Molecular Sciences, 23(7), 3501. 10.3390/ijms23073501 35408868 PMC8998961

