# Supplementary material for: Circulating extracellular vesicle characteristics differ between men and women following 12 weeks of concurrent exercise training
Source: Physiol Rep. 2024 May 2;12(9):e16016. doi: 10.14814/phy2.16016 (PMC11065700; doi:10.14814/phy2.16016)
Supplement: Supplementary file 1 — Table S1. Table S2. Table S3. [file PHY2-12-e16016-s003.docx]

**Supplementary Table 1: Exercise training program description.**

| Mesocycle | Duration (weeks) | Set Range | Repetition Range | Intensity (% 1RM) |
| --- | --- | --- | --- | --- |
| General Physical Preparedness | 2 | 3 | 10 | 64 - 72% |
| Preparation for Peak Force Production | 1 | 3 – 4 | 5 – 6 | 72 - 80% |
| Peak Force Development | 3 | 3 - 5 | 3 | 79 - 88% |
| Rate of Force Development | 3 | 3 - 4 | 2 – 3 | 81 - 90% |

**Supplementary Table 2: Number of Differentially expressed miRNAs and subsequent number of IPA identified mRNA targets.**

| Comparison | Number of Differentially Expressed miRNAs | Targeted mRNAs identified by IPA |
| --- | --- | --- |
| Male: Baseline: Resting vs Post Acute Exercise | 33 | 1721 |
| Female Baseline: Resting vs Post Acute Exercise | 42 | 1365 |
| Male Trained: Resting vs Post Acute Exercise | 7 | 666 |
| Female Trained: Resting vs Post Acute Exercise | 36 | 1297 |
| Male Resting: Pre vs Post Chronic Training | 39 | 1742 |
| Female Resting: Pre vs Post Chronic Training | 11 | 733 |
| Male Post Acute Exercise: Pre vs Post Chronic Training | 7 | 415 |
| Female Post Acute Exercise: Pre vs Post Chronic Training | 32 | 1577 |

**Supplementary Table 3: Demographic information for subset of subjects who had EV miRNA sequencing performed.**

|  | **Men**  **(*n* = 5)** | | **Women**  **(*n* = 5)** | |
| --- | --- | --- | --- | --- |
|  | Baseline | 12 weeks | Baseline | 12 weeks |
| **Age (y)** | 25.8 ± 4.6  (18 – 30) | | 28.0 ± 4.9  (23.0 – 36.0) | |
| **Height (cm)*** | 179.1 ± 2.6  (175.5 − 181.6) | | 166.1 ± 6.9  (157 − 174.6) | |
| **Body mass (kg)*** | 83.8 ± 6.4  (77.1 – 92.4) | 85.2 ± 5.5  (79.3.1 – 93.6) | 66.5 ± 10.5  (52.3 – 79.1) | 67.7 ± 9.9  (53.9 – 78.27) |
| **Body fat (%)*** | 23.8 ± 5.0  (16 – 28.9) | 22.8 ± 4.4^p=0.057^  (15 – 26.8) | 29.6 ± 2.7  (25.2 – 31.6) | 28.9 ± 3.1#  (23.8 – 31.5) |
| **Total Lean Mass mass (kg)*** | 60.7 ± 4.3  (55.5 – 67.8) | 63.0 ± 5.1^#^  (57.5 – 69.8) | 44.5 ± 6.7  (37.0 – 54.0) | 45.8 ± 6.2^p=0.057^  (39.2 – 53.4) |

*Indicates a significant difference between men and women. #Indicates a significant difference between pre- and post-training. Data are presented as mean ± standard deviation (range). Significance was set at *p* < 0.05.
