# Supplementary material for: Circulating extracellular vesicle characteristics differ between men and women following 12 weeks of concurrent exercise training
Source: Physiol Rep. 2024 May 2;12(9):e16016. doi: 10.14814/phy2.16016 (PMC11065700; doi:10.14814/phy2.16016)

Samples were loaded in duplicates that are side by side. Loading order for each gel is as follows: Ladder, Baseline Resting (Lane 3-4), Baseline Post-Exercise (Lane 5-6), Trained Resting (Lane 7-8), Trained Post-exercise (Lane 9-10). The representative images in the manuscript have been cropped to remove duplicates and to only include Resting Akt values. No other modifications have been made to the images.

For the manuscript, only resting values (Baseline and Trained) were used for analysis in order to determine the impact of chronic exercise on t-Akt

t-Akt blot is shown on the left side and stain free imaging of total protein on the right side. Stain free total protein images of the membranes were used for loading normalization. Representative images have been taken from Male 1 and Female 2.

Male 1:


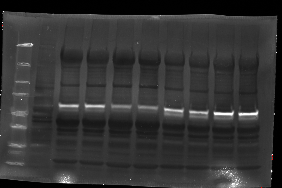

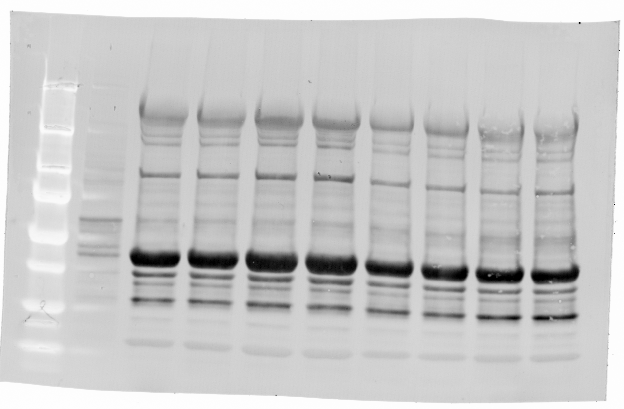


Male 2:


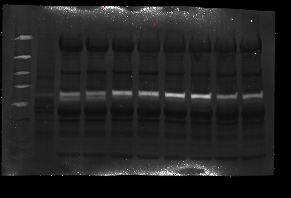

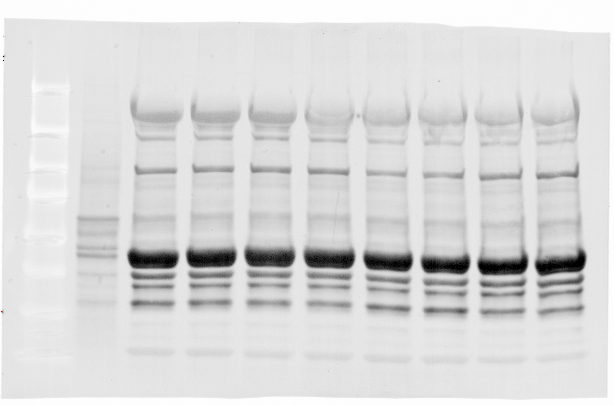


Male 3:


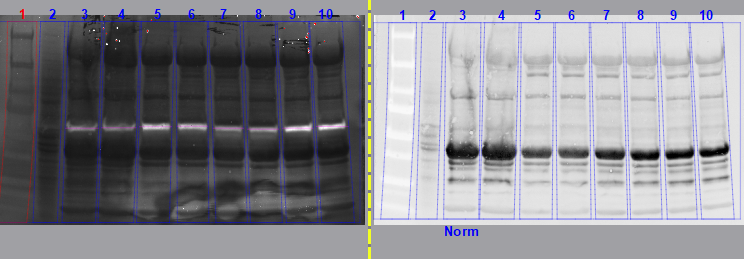


Male 4:
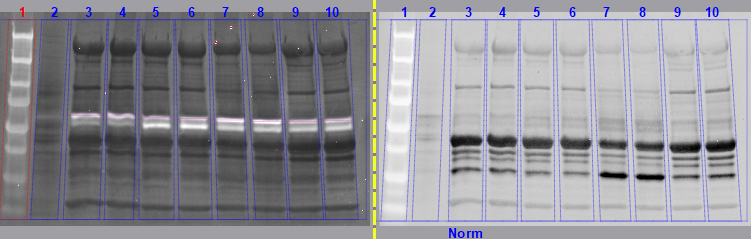


Male 5:


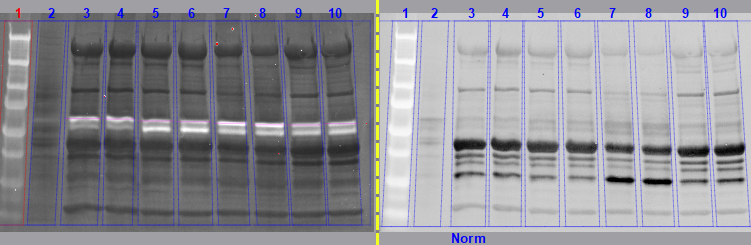


Female 1:


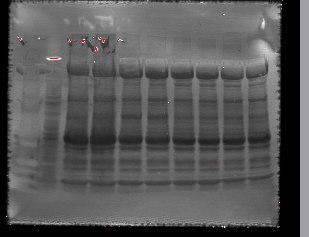

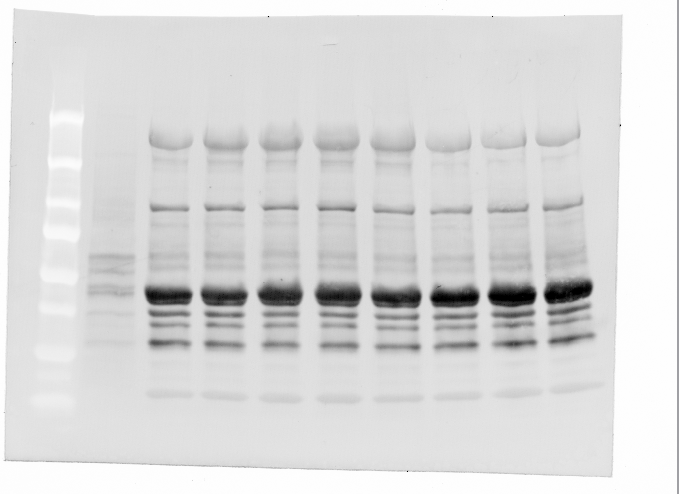


Female 2:


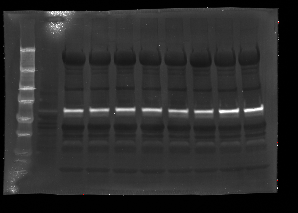

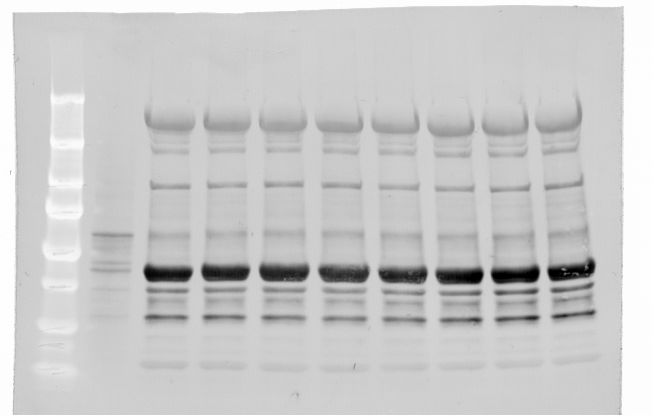


Female 3:
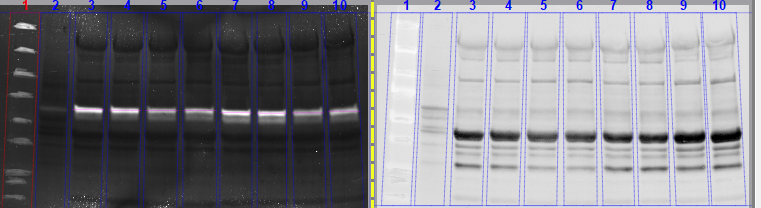


Female 4:


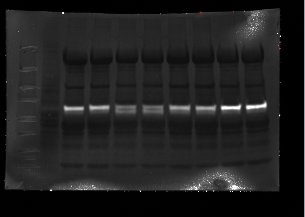

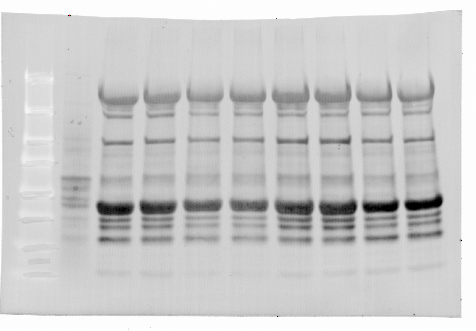


Female 5:


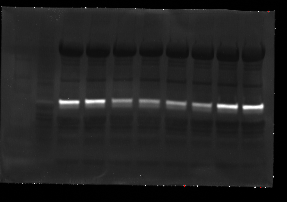

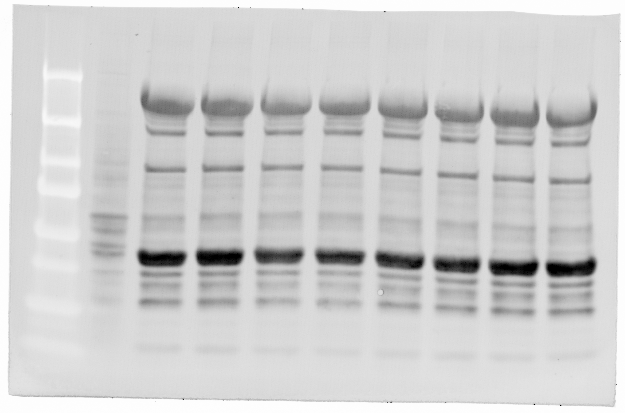

Supplement: Supplementary file 2 — Data S1. [file PHY2-12-e16016-s005.docx]
